# Rehabilitation at the Time of Pandemic: Patient Journey Recommendations

**DOI:** 10.3389/fnagi.2022.781226

**Published:** 2022-04-12

**Authors:** Ahmed M. Negm, Adrian Salopek, Mashal Zaide, Victoria J. Meng, Carlos Prada, Yaping Chang, Preeti Zanwar, Flavia H. Santos, Elena Philippou, Emily R. Rosario, Julie Faieta, Shanti M. Pinto, Jason R. Falvey, Amit Kumar, Timothy A. Reistetter, Vanina Dal Bello-Haas, Mohit Bhandari, Jonathan F. Bean, Patricia C. Heyn

**Affiliations:** ^1^Faculty of Rehabilitation Medicine, University of Alberta, Edmonton, AB, Canada; ^2^School of Rehabilitation Science, McMaster University, Hamilton, ON, Canada; ^3^Faculty of Health Sciences, McMaster University, Hamilton, ON, Canada; ^4^Faculty of Sciences, McMaster University, Hamilton, ON, Canada; ^5^Division of Orthopedic Surgery, Department of Surgery, McMaster University, Hamilton, ON, Canada; ^6^OrthoEvidence Inc., Burlington, ON, Canada; ^7^Center for Population Health & Aging, Center for Health Systems & Design, Texas A&M University, College Station, TX, United States; ^8^U.S. Network on Life Course and Health Dynamics & Disparities, College Station, TX, United States; ^9^School of Psychology, U.C.D. Centre for Disability Studies, University College Dublin, Dublin, Ireland; ^10^Department of Life and Health Sciences, School of Sciences and Engineering, University of Nicosia, Nicosia, Cyprus; ^11^Department of Nutritional Sciences, King’s College London, London, United Kingdom; ^12^Casa Colina Hospital and Centers for Healthcare, Pomona, CA, United States; ^13^Department of Rehabilitation Science and Technology, University of Pittsburgh, Pittsburgh, PA, United States; ^14^Department of Physical Medicine and Rehabilitation, Carolinas Rehabilitation, Charlotte, NC, United States; ^15^Department of Physical Therapy and Rehabilitation Science, University of Maryland School of Medicine, Baltimore, MD, United States; ^16^Department of Epidemiology and Public Health, University of Maryland School of Medicine, Baltimore, MD, United States; ^17^College of Health and Human Services, Northern Arizona University, Flagstaff, AZ, United States; ^18^Department of Occupational Therapy, School of Health Professions, University of Texas Health Science Center at San Antonio, San Antonio, TX, United States; ^19^Department of Surgery, Faculty of Health Sciences, McMaster University, Hamilton, ON, Canada; ^20^Department of PM&R, New England Geriatric, Research, Education and Clinical Center, VA Boston Healthcare System, Boston, MA, United States; ^21^Spaulding Rehabilitation Hospital, Harvard Medical School, Boston, MA, United States; ^22^Marymount Center for Optimal Aging, School of Health Sciences, Marymount University, Arlington, VA, United States

**Keywords:** COVID-19, pandemic, rehabilitation, scoping review, GRADE, physiotherapy, occupational therapy, ICU rehabilitation

## Abstract

**Purpose:**

The World Health Organization (WHO) declared severe acute respiratory syndrome coronavirus 2 (SARS-CoV-2) a pandemic in March 2020, causing almost 3.5 million coronavirus disease (COVID-19) related deaths worldwide. The COVID-19 pandemic has imposed a significant burden on healthcare systems, economies, and social systems in many countries around the world. The access and delivery of rehabilitation care were severely disrupted, and patients have faced several challenges during the COVID-19 outbreak. These challenges include addressing new functional impairments faced by survivors of COVID-19 and infection prevention to avoid the virus spread to healthcare workers and other patients not infected with COVID-19. In this scoping review, we aim to develop rehabilitation recommendations during the COVID-19 pandemic across the continuum of rehabilitation care.

**Materials and Methods:**

Established frameworks were used to guide the scoping review methodology. Medline, Embase, Pubmed, CINAHL databases from inception to August 1, 2020, and prominent rehabilitation organizations’ websites were searched.

**Study Selection:**

We included articles and reports if they were focused on rehabilitation recommendations for COVID-19 survivors or the general population at the time of the COVID-19 pandemic.

**Data Extraction:**

Two of our team members used the pre-tested data extraction form to extract data from included full-text articles. The strength and the quality of the extracted recommendations were evaluated by two reviewers using the GRADE (Grading of Recommendations, Assessment, Development and Evaluation) approach.

**Results:**

We retrieved 6,468 citations, of which 2,086 were eligible after removing duplicates. We excluded 1,980 citations based on the title and the abstract. Of the screened full-text articles, we included 106 studies. We present recommendations based on the patient journey at the time of the pandemic. We assessed the evidence to be of overall fair quality and strong for the recommendations.

**Conclusion:**

We have combined the latest research results and accumulated expert opinions on rehabilitation to develop acute and post-acute rehabilitation recommendations in response to the global COVID-19 pandemic. Further updates are warranted in order to incorporate the emerging evidence into rehabilitation guidelines.

## Introduction

SARS-n-CoV-2 (a novel coronavirus), causing severe respiratory disease, was first formally identified in Wuhan City, China, on December 31, 2019, and within a few months spread globally World Health Organization [WHO] (2020). The World Health Organization (WHO) declared the disease caused by the novel coronavirus COVID-19, a pandemic on March 11, 2020. COVID-19 has impacted nations worldwide, regardless of climate, population, and location World Health Organization [WHO] (2020). The impact of the pandemic has been devastating and long-lasting, not only to health and healthcare systems but to social systems and economies as well, with subsequent burdens at the community level.

In order to prevent the spread of the disease, the general population has been impacted through restrictions that have limited access to primary care, elective and non-elective surgeries, urgent care, outpatient rehabilitation, and post-acute rehabilitation, both in patients with COVID-19 and without COVID-19 infection. Frail older adults are at the greatest risk for severe complications and mortality after COVID-19 infection due to age-related comorbidities (such as diabetes, hypertension, and frailty), which impair their ability to fight severe COVID-19 related pneumonia (Meftahi et al., 2020; Perrotta et al., 2020; Stawicki et al., 2020; United Nations, 2020). Data collected in South Korea, Italy, France, Germany, England, and Spain indicate that the mortality rate from COVID-19 infection increased by 12% per year after the age of 70-years (Goldstein and Lee, 2020).

Rehabilitation is essential after recovery from numerous health conditions such as acute stroke (Smith et al., 2020), cardiac events, and infectious diseases (Besnier et al., 2020; Scherrenberg et al., 2020). The pandemic has led to frequent cancelation of elective surgeries, a step taken with the aim of decreasing hospital utilization, preserving Intensive Care Unit (I.C.U.) capacity, conserving Personal Protective Equipment (P.P.E.) and allowing for redeployment of healthcare workers to care for those with COVID-19 infection. As a result, rehabilitation services are in higher demand since the beginning of the global pandemic. However, the rehabilitation community has faced a number of challenges in the context of COVID-19. These challenges include: (1) Addressing multifactorial functional impairments seen among COVID-19 survivors because of lung, heart, kidney, vascular endothelium, muscular and central nervous system effects of the disease (Centre for Disease Control Prevention, 2021); (2) Infection prevention to avoid virus spread to healthcare workers and other patients not infected with COVID-19; (3) Provision of acute rehabilitation; (4) Provision of post-acute rehabilitation after discharge from acute hospital, and (5) Transitioning to a telehealth care-delivery model. Adaptations were necessary to: facilitate care for a population with complex medical and functional impairments (COVID-survivors); prevent infection; preserve P.P.E., and accommodate facility and/or local policies to mitigate the spread of COVID-19 (Levi et al., 2020; Miles et al., 2020; Salawu et al., 2020).

A large number of COVID-19 patients ended up hospitalized and admitted to the intensive care unit. Many of these patients ended up on ventilator machines and intubated, ultimately impacting speech and swallowing function, respiratory function, and overall physical function, and these patients will be benefited from timely and comprehensive rehabilitation care. But, there is a paucity of evidence on rehabilitation recommendations during COVID-19 (Simpson and Robinson, 2020) which may contribute to high rates of unmitigated disability following coronavirus infections. Rooney et al. (2020) Clinic closures and restrictions placed on rehabilitation personnel entering certain high-risk facilities have contributed to a substantial reduction in rehabilitation volume delivered to frail older adults (Falvey et al., 2020). There is clear evidence that rehabilitation services are associated with improvements in physical and cognitive function (Zhang et al., 2019). Therefore, failure to provide rehabilitation services may leave patients vulnerable to avoidable hospitalizations (such as those from fall-related trauma), worsening disability, higher caregiver burden, and lower quality of life. For individuals with frailty, the negative impacts of reduced rehabilitation service may be magnified given their higher vulnerability to functional decline (Ferrante et al., 2018), leading to further declines in functional reserve and increased vulnerability to adverse events (Hosey and Needham, 2020).

Thus, there is an urgent need to develop rehabilitation recommendations that aim to provide guidance to rehabilitation institutions and professionals on safe and effective practices across the continuum of rehabilitation care during the COVID-19 pandemic (Thornton, 2020). These recommendations can be used to help inform the public that rehabilitation is an essential medical intervention that has been poorly prioritized during the pandemic, leading to unnecessary suffering and added disability burden for those infected with COVID-19, as well as those who have been unable to receive needed care because of COVID-19 related restrictions. In response to the global pandemic, we launched a COVID task force in the American Congress of Rehabilitation Medicine (ACRM) to help address the lack of contemporary research assessing the impact of COVID-19 on rehabilitation. The task force comprises a multidisciplinary team of clinicians and researchers with a diversity of rehabilitation and health services expertise across the continuum of rehabilitation settings. The purpose of this paper is to develop rehabilitation recommendations during the COVID-19 pandemic across the continuum of rehabilitation care.

## Materials and Methods

We used the framework proposed by Arksey and O’Malley (2005) and Levac et al. (2010) to guide the scoping review methodology. We followed the Preferred Reporting Items for Systematic Reviews and Meta-Analyses (PRISMA) Extension for Scoping Reviews (PRISMA-ScR) guidelines to warrant a high quality of research reporting (Tricco et al., 2018).

### Development of Research Questions

The main concepts of interest are the COVID-19 pandemic and rehabilitation (including physiotherapy, physical therapy, occupational therapy, speech-language pathology, physiatry, and other rehabilitation services). The outcomes of interest were rehabilitation recommendations based on research data, consensus, or expert opinions.

### Identifying Relevant Studies

A health sciences librarian developed and implemented literature searches in Medline, Embase, Pubmed, CINAHL, and gray literature including major rehabilitation websites/organizations from inception to August 1, 2020. Our multidisciplinary study members helped conceptualize the search strategy (which was based on the concepts of the COVID-19 pandemic and rehabilitation) with multiple text words and subject headings (e.g., MeSH) describing each concept. This search strategy was limited to English. The search strategies are detailed in the Supplementary Materials.

### Selection Criteria

Studies were included if they discussed rehabilitation recommendations for COVID-19 patients, survivors, or the general population at the time of the COVID-19 pandemic.

### Screening and Study Selection

Search results were uploaded to the Covidence platform (Covidence Systematic Review Software, 2021). After removing duplicates, two of four team members (MZ, AS, VM, AN) independently reviewed the titles and abstracts following the inclusion/exclusion criteria. If there were insufficient details to make an informed decision, the article was retrieved for review. To confirm eligibility, two of four team members (MZ, AS, VM, AN) independently assessed the full-text articles using the same inclusion criteria. Any disagreement was resolved through consensus or third-party adjudication by a senior reviewer (AN).

### Data Extraction

A standardized data extraction form was created by the research team. Two of four team members (MZ, AS, VM, AN) then used the pre-tested data extraction form to extract data from included full-text articles.

### Quality Assessment

**FIGURE 1:**
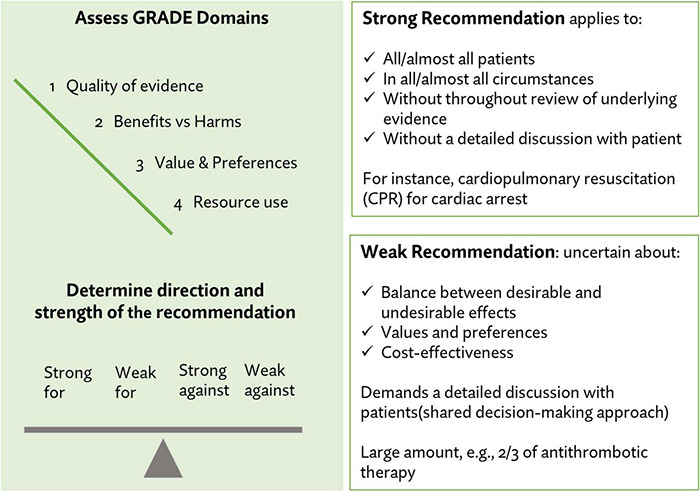
Strength and quality assessments for individual recommendations (Guyatt et al., 2008).

Two reviewers (MZ, VM) evaluated the strength and the quality of the extracted recommendations using the GRADE (Grading of Recommendations, Assessment, Development and Evaluation) approach of selected full-text articles using the Oxford Level of Evidence (Guyatt et al., 2008). There are four possible categories for strength of recommendation evidence: (1) strong recommendation for; (2) weak recommendation for; (3) weak recommendation against, and (4) strong recommendation against. Figure 1 shows the GRADE strength categories and outlines the clinical application of recommendations based on level of strength. There are three categories for quality of recommendation: (1) Good, (2) Fair, and (3) Poor. We present the quality and strength of key recommendations throughout the results of the review.

### Summarizing and Reporting the Findings

The extracted recommendations were organized into several sections. These sections were decided with input from coauthors (group of experts in rehabilitation sciences). We reported a brief summary of the studies along with the strength and the quality of recommendations.

## Results

**FIGURE 2:**
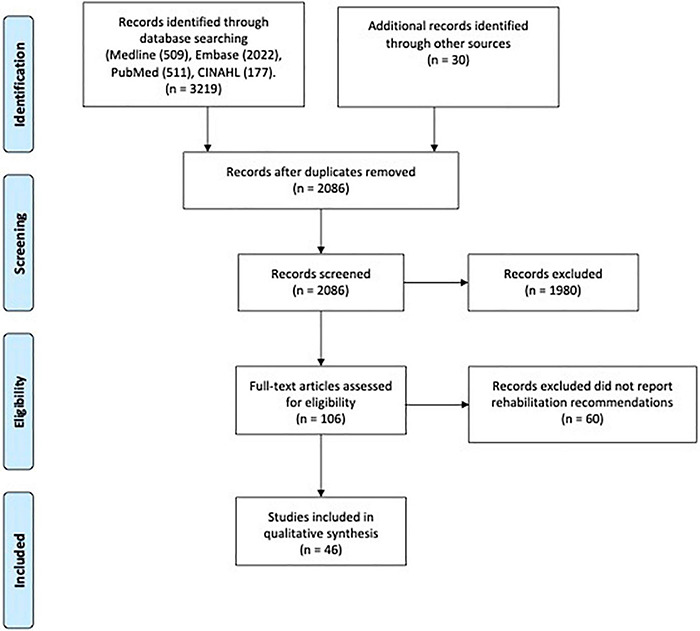
PRISMA flow diagram.

Of the 6,468 citations retrieved, 2,086 were eligible for screening after removing duplicates. We excluded 1,980 after the title and abstract screening. Of the screened full-text articles, 106 studies from 22 countries (including low-income, middle-income and high-income) reported COVID-19 related recommendations (Figure 2). Of these articles, 46 articles reported rehabilitation recommendations across patients’ journeys. A reference list of the 46 articles is provided in the Supplementary Materials.

### The Extracted Recommendations

**FIGURE 3:**
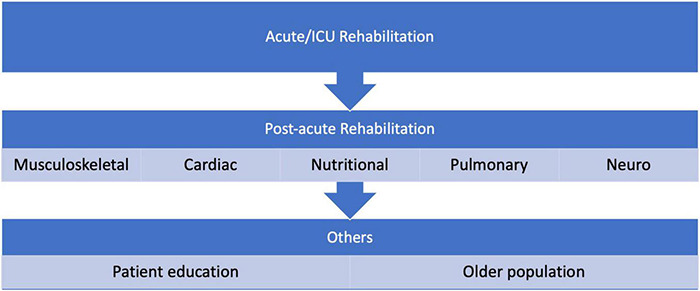
Structure of patient journey recommendations.

In this guideline, we present recommendations focused on the patient journey at the time of the pandemic. Another manuscript presented the health system-related recommendations (Negm et al., 2022). Figure 3 summarizes the structure of the recommendation.

### Quality and Strength of the Recommendations

Using the GRADE approach to evidence quality assessment, we assessed the evidence to be overall of fair quality and strong for the recommendations made (Table 1). The strength of each individual recommendation is reported in the Supplementary Table.

**TABLE 1:** Recommendations quality.

Standard	Rating
Establishing transparency	Fair
Management of C.O.I.* in the guideline development group	Fair
Recommendation development group composition	Good
Recommendation development (evidence-based)	Fair
Establishing evidence foundations and rating strength for each of the recommendations	Fair
Articulation of recommendations	Fair
External review	Not Reported
Updating	Fair
Implementation issues	Not Reported

* C.O.I., Conflict of interest.

### Acute Care/I.C.U. Rehabilitation

We identified 17 publications that addressed the acute or I.C.U. rehabilitation domain. Publication dates ranged from March 30, 2020, to August 1, 2020 (Amatya and Khan, 2020; Arenivas et al., 2020; Chinese Association of Rehabilitation Medicine et al., 2020; Hosey and Needham, 2020; Kalirathinam et al., 2020; Masiero et al., 2020; Nakamura et al., 2020; Pinto and Carvalho, 2020; Qu et al., 2020; Recommendations AHSSAGC, 2020; Sañudo et al., 2020; Sheehy, 2020; Thomas et al., 2020; Vitacca et al., 2020; Yu P. et al., 2020; Handu et al., 2021; Kurtais Aytür et al., 2021). Recommendations were from 11 countries [Canada (*n* = 3), Australia (*n* = 2), Brazil (*n* = 1), China (*n* = 3), Italy (*n* = 2), Japan (*n* = 1), Belgium (*n* = 1), Spain (*n* = 1), Turkey (*n* = 1), United Kingdom (*n* = 1), United States (*n* = 3), one study included data from multiple countries (Australia, Belgium, Canada)]. Seventeen institutions participated in developing these guidelines, including hospitals, scientific societies, and universities (Supplementary Table). Of the 17 publications, eight were developed by rehabilitation or medical professions, and five were developed by researchers. The other four publications did not report the group involved in developing the recommendations. Of the 17 publications, 16 were developed based on expert opinion and/or clinical experience, and a single study was developed based on a combination of both evidence-based methods and expert opinion.

### Summary of Key Recommendations

• In patients with COVID-19 pneumonia or acute respiratory distress syndrome (ARDS), include a multidisciplinary/holistic care program for pulmonary rehabilitation tailored to the unique needs of each patient, giving meticulous attention to the delivery of evidence-based critical care interventions, to early comprehensive rehabilitation that targets physical and neuropsychological recovery, and for the evaluation of adequate social support.
• If indicated, use airway clearance techniques including positioning, active breathing cycle, manual and ventilator hyperinflation, percussion and vibrations, positive expiratory pressure therapy (P.E.P.), assisted or stimulated cough maneuvers, airway suctioning, and mechanical insufflation-exsufflation.
• Positioning management: When physiological status permits, gradually change positioning in more vertical anti-gravity postures, such as raising the bed head.
• Carry out positioning management in 30-min sessions, three sessions per day. Prone position ventilation is implemented in patients with ARDS for 12 h and above.
• In the supine position, place the lower edge of the pillow on one-third of the scapula to prevent head hyperextension and place a pillow below the popliteal fossa to relax the lower limbs and abdomen.
• Ensure safety and integrity of tubing and lines to prevent inadvertent disconnection or detachment during mobilization and rehabilitation interventions.
• Monitor vital signs during rehabilitation sessions and vital sign responses to rehabilitation interventions.
• Consider intensity and duration of rehabilitation activities and sessions and adjust accordingly for patients with poor physical status.
  ◦ Lower intensity and shorter duration of activities, exercises, with sessions only for patients with poor physical status.
  ◦ Keep single rehabilitation sessions to less than 30 min to reduce fatigue.
  ◦ Passive, active-assisted, active range of motion or resistance exercises may be performed to maintain or improve joint integrity, range of motion, and muscle strength.
• Mobility/mobilization/exercises may include side-to-side position changes, bed mobility, sitting at the bed edge, moving from the bed to chair, sitting in a chair, standing, stepping in place, walking, tilt table, standing hoists, upper/lower cycle ergometry, and exercise programs; and active range of motion (R.O.M.) exercises through the full available range.
• The treatment plan for patients receiving sedatives or unconscious includes in-bed cycling, passive R.O.M. exercises, stretching exercises, and neuromuscular electrical stimulation.
• Indicate reconditioning interventions in weaned patients and those with prolonged weaning from mechanical ventilation and oxygen use to improve physical function and capacity and address motor and cognitive effects of prolonged immobilization in I.C.U.
• Neuropsychologists assess and treat varied cognitive presentations in acute rehabilitation settings. Build cognitive flexible evaluation methods that can be adapted to the patient’s functional level.
• Utilize neuropsychologists to provide emotional support and use evidence-based interventions to promote mental health and coping skills.
• In individuals with suspected or confirmed COVID-19 infection in the I.C.U. who are not mechanically ventilated, registered dietitians (RD) should work with the multidisciplinary team to ensure adequate energy and protein intake.
• When needs cannot be met orally, enteral nutrition (EN) is the preferred feeding route. If EN is not suitable or accepted/has to be initiated in a timely manner to treat and prevent any further malnutrition.

### Post-acute Rehabilitation

We identified 25 publications that addressed post-acute rehabilitation. Publication dates ranged from February 4 to July 5, 2020 (Barker-Davies et al., 2020; Bartolo et al., 2020; Bij de Vaate et al., 2020; Boldrini et al., 2020; Brugliera et al., 2020; Chen et al., 2020; Chinese Association of Rehabilitation Medicine et al., 2020; Galiuto and Crea, 2020; Gómez-Moreno et al., 2020; Jangra and Saxena, 2020; Kho et al., 2020; Mammi et al., 2020; Pandian and Sebastian, 2020; Piepoli, 2020; Polastri et al., 2020; Qu et al., 2020; Sañudo et al., 2020; Schmidt et al., 2020; Sheehy, 2020; Vitacca et al., 2020; Wade, 2020; Yang and Yang, 2020; Yu H.P. et al., 2020; Zhao et al., 2020; Handu et al., 2021; Kurtais Aytür et al., 2021). Recommendations were from 12 countries [Canada (*n* = 2), China (*n* = 5), India (*n* = 2), Italy (*n* = 8), Mexico (*n* = 1), Denmark (*n* = 1), United States (*n* = 2), Netherlands (*n* = 1), Portugal (*n* = 1), Spain (*n* = 1), Turkey (*n* = 1), United Kingdom (*n* = 2), one study included data from multiple countries (China, Denmark, United States)]. Twenty-five institutions participated in developing these guidelines including hospitals, scientific societies, and universities (Supplementary Table).

Of the 25 publications, nine were developed by rehabilitation or medical professions, and eight were developed by researchers. Other (Besnier et al., 2020) publications did not report the group involved in developing the recommendations. Of the 25 publications, 22 were developed based on expert opinion and/or clinical experience, two were developed using evidence-based methods including systematic review, survey, and observational studies, and one study was developed based on a combination of both evidence-based methods and expert opinion.

### Summary of Key Recommendations

**A) Neurorehabilitation**

• The role of occupational therapists or healthcare professionals with similar training should include:
  ◦ Prevention, detection, and monitoring of delirium.
  ◦ Assessment and management of impairments in physical and cognitive functioning.
  ◦ Evaluation of emotional coping strategies for patients.
  ◦ Addressing mental health and psychosocial needs of patients and/or caregivers.
• Inpatient rehabilitation settings: Ensure the adequate delivery of interventions and development of individual rehabilitation plans for patients directly admitted from the acute care wards, including patients recovering from COVID-19 with disabling sequelae.
• Patients with neurological conditions requiring rehabilitation coming from acute units outside hospitals should be admitted if tested as COVID-19 negative using throat and nasal swabs and after confirming absence of fever and cough (or other symptoms suggestive of COVID-19 infection).

Use psychosocial support to manage emotional disturbance, changes in self-esteem and self-confidence, and similar constructs with techniques such as cognitive-behavioral therapy and motivational interviewing (Bartolo et al., 2020; Chinese Association of Rehabilitation Medicine et al., 2020; Kho et al., 2020; Pandian and Sebastian, 2020; Qu et al., 2020; Sheehy, 2020; Wade, 2020).

**B) Nutritional Rehabilitation and Speech Therapy**

• Early assessment of nutritional status with consequent addition of energy and protein through oral food supplements, or if not tolerated, transition to artificial nutrition.
• Speech-Language Pathology role should include:
  ◦ Assessment and management post-extubation dysphagia upon decompensation and respiratory compromise.
  ◦ Assessment of basic cognitive and communication functions.
  ◦ Assessment and treatment of voice impairments caused by prolonged intubation.
• Implement early nutritional supplement protocol for non-critical COVID-19 patients with severe inflammatory status and anorexia, which can lead to a significant reduction in food intake.
• When counseling patients with suspected or confirmed COVID-19 infections who are in their homes or the outpatient setting, R.D.s’ advice to patients and their families should include the following:
  ◦ Adequate energy and protein intake by meeting at least the recommended dietary allowance for energy and protein-based on age and sex.
  ◦ If the oral dietary intake is inadequate, nutrient-dense foods and beverages, including oral nutritional supplements, should be recommended to increase energy and protein intake.
  ◦ Beverages should be recommended to increase energy intake; if an individual is unable to eat solid foods due to difficulty coordinating chewing and breathing.
  ◦ Micronutrient supplements help counteract for inadequate oral intake to address deficiencies.
  ◦ Small frequent meals and snacks should be recommended to avoid nausea, vomiting, and shortness of breath.
  ◦ Provide foods that require little handling, preparation, or effort to eat.

Adequate intake of fluids to stay hydrated throughout the day and evening. Use rehydration drinks if the patient is experiencing vomiting and diarrhea (Brugliera et al., 2020; Chinese Association of Rehabilitation Medicine et al., 2020; Kho et al., 2020; Sheehy, 2020; Handu et al., 2021).

**C) Musculoskeletal and Cardiorespiratory Rehabilitation**

• Standardized rehabilitation evaluation including:
  ◦ Clinical evaluation: physical examination, imaging tests, laboratory tests, lung function tests, nutrition screening, and ultrasonography.
  ◦ Exercise and respiratory function assessment: (i) respiratory muscle strength: maximum inspiratory pressure/maximum expiratory pressure; (ii) muscle strength: manual muscle testing using the Medical Research Council scale; or isokinetic muscle testing; (iii) joint R.O.M. test; (iv) balance function evaluation: Berg Balance Scale; (v) aerobic exercise capacity: 6-min walk test and cardiopulmonary exercise testing; and (vi) physical activity evaluation: International Physical Activity Questionnaire and Physical Activity Scale for the Elderly.
  ◦ Evaluation of activities of daily living (A.D.L.): The Barthel index or equivalent instruments.
• The role of occupational therapists or healthcare professionals with similar training should include:
  ◦ Optimizing bed and seating positioning using pressure relief principles (e.g., mattress).
  ◦ Assessment and management of A.D.L.s and instrumental activities of daily living (IADLs) to encourage early mobilization.
  ◦ Provision of assistive devices for A.D.L.s, communication, seating, and mobility.
  ◦ Facilitate functional independence/autonomy and preparing patients for discharge.
• The role of physical therapy or healthcare professionals with similar training includes:
  ◦ Assessment of exercise and functional capacity.
  ◦ Monitoring of pre-existing comorbid conditions.
  ◦ Exercise training and/or physical activity coaching.
  ◦ Enhance mobility, A.D.L., and IADL.
• According to the cognitive and emotional dysfunction level, an individual can choose a first-line physical therapy under general practitioner supervision or an integrated treatment program in a COVID-19 rehabilitation clinic (if available).
• Aerobic exercises are tailored based on the patient’s underlying COVID-19 disease and remaining dysfunction. Aerobic exercises, such as walking, slow jogging, and swimming, should begin at a low intensity then gradually increase. A total of 3 to 5 sessions should be carried out every week, each session lasting 20–30 min. Patients should use intermittent exercises if they are prone to fatigue.
• Progressive resistance training is recommended for strength training: 8 to 12 repetitions per set, 1 to 3 sets for each target muscle group, with 2-min rest intervals between sets, at a frequency of 2 to 3 sessions/week for six weeks.
• Patients with comorbid balance disorders should perform balance training.
• Adjust the exercise program plan to accommodate the patients’ home environment. Help patients identify safe, alternative spaces for aerobic training according to current government/jurisdictional COVID-19 guidelines.
• Use COVID-19 illness severity to determine the exercise intensity:
  ◦ For mild COVID-19 illness, (i) Exercise intensity measured by Modified BORG Dyspnea Scale ≤ 3 points; (ii) Exercise frequency should be twice a day, duration 15–45 min each session, 1 h after meals; and (iii) examples of types of exercise include Tai chi, breathing exercise, or square dancing.

For moderate COVID-19 illness, (i) Exercise intensity should be between rest [1.0 metabolic equivalents (M.E.T.s)] and light exercise (< 3.0 M.E.T.s); (ii) Exercise frequency should be twice a day, 1 h after a meal, duration should be based on the individual’s physical status, and each session lasts 15–45 min. Individuals should perform intermittent exercise if they are prone to fatigue; (iii) examples of types of exercise include stepping, Tai chi, breathing exercises (Barker-Davies et al., 2020; Bij de Vaate et al., 2020; Chen et al., 2020; Chinese Association of Rehabilitation Medicine et al., 2020; Gómez-Moreno et al., 2020; Kho et al., 2020; Mammi et al., 2020; Piepoli, 2020; Qu et al., 2020; Schmidt et al., 2020; Sheehy, 2020; Vitacca et al., 2020; Wade, 2020; Yu H.P. et al., 2020).

**D) Pulmonary/Cardiorespiratory Rehabilitation**

• Respiratory assessment should include evaluation of dyspnea, thoracic activity, diaphragmatic activity and amplitude, respiratory muscle strength (maximal inspiratory and expiratory pressures), respiratory pattern, and frequency. Cardiac status should also be assessed.
• Determine respiratory rehabilitation goals
  ◦ The short-term goal of pulmonary rehabilitation should be to lessen dyspnea and reduce anxiety and depression.
  ◦ The long-term goal of pulmonary rehabilitation should be to reserve the patient’s function, improve quality of life, and accelerate return to the community.
• Primary intervention measures for pulmonary rehabilitation include airway clearance, reduction of dyspnea, breathing control, physical activity, and exercise.
• Airway clearance techniques: in patients with chronic airway disease, forced expiratory techniques should be used in the early stages of airway clearance after discharge to expel sputum and reduce coughing and energy consumption; positive expiratory pressure/OPEP can be used as aids.
• Breathing control: (i) positioning: An upright sitting position. If a patient has shortness of breath, a semi-sitting position should be used; (ii) maneuvers: the patient slowly inhales through the nose and slowly exhales through the mouth while the shoulders and neck accessory muscles are relaxed.
• In the post-acute phase, inspiratory muscle training should be included if inspiratory muscles are weak.
• Two sessions of 10 min of pulmonary rehabilitation every week for six weeks following discharge from acute care showed a significant improvement in pulmonary function, endurance, quality of life, and depression.

Breathing exercise: if shortness of breath, wheezing, and difficulty in expelling sputum occur in patients after discharge, breathing exercise (such as posture management, adjustment of breathing rhythm, thoracic expansion training and mobilization of respiratory muscle groups) should be used (Chinese Association of Rehabilitation Medicine et al., 2020; Jangra and Saxena, 2020; Polastri et al., 2020; Qu et al., 2020; Sañudo et al., 2020; Sheehy, 2020; Vitacca et al., 2020; Yang and Yang, 2020; Zhao et al., 2020; Kurtais Aytür et al., 2021).

**E) Cardiac Rehabilitation**

• Give cardiovascular protection during COVID-19 infection and prescribe adequate cardiac rehabilitation programs to survivors of the disease.
• Home-based cardiac rehabilitation (C.R.) programs should comprise the same main components as center-based programs.
• Telemonitoring should include technology-assisted assessments, which range from using a logbook and structured telephone calls to the use of wearable sensors, such as heart rate monitors, accelerometers, or remote E.C.G. telemetry monitoring.
• Patient-related factors (cardiovascular risk, digital skills and personal preferences) and provider-related factors (such as logistical conditions, including staff training and availability of technological equipment) determine the approach and the degree of technological sophistication to use for telemonitoring.
• The video conferencing technologies is used to allow patients to meet with the cardiac rehabilitation team. Video conferencing is a useful tool to decrease the mental and physical consequences of social isolation caused by the COVID-19 pandemic.

A hybrid approach is recommended if patients’ safety is a concern. The hybrid approach begins with supervised sessions in a cardiac rehabilitation unit; the sessions start with a low-intensity exercise prescription to promote patients’ confidence and adherence, followed by weekly telephone calls to discuss exercise progression and any concerns (Galiuto and Crea, 2020; Schmidt et al., 2020).

### Education/Social Interventions

We identified eight publications that addressed education and social interventions (Barker-Davies et al., 2020; Chinese Association of Rehabilitation Medicine et al., 2020; Griffin, 2020; Jalali et al., 2020; Qu et al., 2020; Sheehy, 2020; Wade, 2020; Kurtais Aytür et al., 2021). Publication dates ranged from April 19 to July 5, 2020. Recommendations were from 5 countries [Canada (*n* = 1), China (*n* = 2), Iran (*n* = 1), Turkey (*n* = 1), United Kingdom (*n* = 3)]. Eight institutions participated in developing these guidelines, including hospitals, scientific societies and universities (Supplementary Table).

Of the eight publications, one was developed by physicians, and two were developed by researchers. Other (United Nations, 2020) publications did not report the group involved in developing the recommendations. Of the eight publications, seven were developed based on expert opinion and/or clinical experience, and a single study was developed based on a combination of both evidence-based methods and expert opinion.

### Summary of Key Recommendations

• Education includes many specific areas: patient self-management; caregivers (family and professional) being taught how to support self-management; caregivers being taught to accelerate practice and/or to provide care safely; caregivers being encouraged to facilitate social integration; teaching patients and others as appropriate, about the disease and its management; and setting expectations for all parties.
• Patient education: (1) Advocacy, videos, and booklets are used to help patients understand the disease and treatment process; (2) patients are encouraged to take regular rest and have sufficient sleep; (3) patients are encouraged to eat a balanced diet; (4) patients are advised to stop smoking.

### Timing of Rehabilitation Services in Persons With COVID-19 Infection

We identified nine publications that addressed the start and stop rehabilitation criteria (Aytür et al., 2020; Chinese Association of Rehabilitation Medicine et al., 2020; Kalirathinam et al., 2020; Kemps et al., 2020; Qu et al., 2020; Sheehy, 2020; Thomas et al., 2020; Vitacca et al., 2020; Yang and Yang, 2020). Publication dates ranged from March 30 to July 16, 2020. Recommendations were from 8 countries [Turkey (*n* = 1), China (*n* = 3), United Kingdom (*n* = 1), Netherlands (*n* = 1), Australia (*n* = 1), Belgium (*n* = 1), Canada (*n* = 2), Italy (*n* = 1), one study included data from multiple countries (Australia, Belgium, Canada)]. Nine institutions participated in developing these guidelines, including hospitals, scientific societies and universities (Supplementary Table).

Of the nine publications, two were developed by researchers and four were developed by rehabilitation and medical professionals. Other (Perrotta et al., 2020) publications did not report the group involved in developing the recommendations. Of the nine publications, eight were developed based on expert opinion and/or clinical experience, and one study was developed based on a combination of both evidence-based methods and expert opinion.

### Summary of Key Recommendations

• **Start Criteria:** Zhao et al. recommended that “In the critically ill COVID-19 patient, respiratory rehabilitation can be initiated once all of the following criteria are met: (1) respiratory system: (i) fraction of inspired oxygen ≤ 0.6, (ii) SpO2 ≥ 90%, (iii) respiratory rate ≤ 40 breaths/min (bpm), (iv) positive end expiratory pressure ≤ 10 cmH2O (1 cmH2O = 0.098 kPa), (v) absence of ventilator resistance, and (vi) absence of unsafe hidden airway problems; (2) cardiovascular system: (i) systolic blood pressure ≥ 90 and ≤ 180 mmHg, (ii) mean arterial pressure (M.A.P.) ≥ 65 and ≤ 110 mmHg, (iii) heart rate ≥ 40 and ≤ 120 beats/min, (iv) absence of new arrhythmia or myocardial ischemia, (v) absence of shock with lactic acid level ≥ 4 mmol/L, (vi) absence of new unstable deep vein thrombosis and pulmonary embolism, and (vii) absence of suspected aortic stenosis; (3) nervous system: (i) Richmond Agitation-Sedation Scale score: −2 to +2 and (ii) intracranial pressure < 20 cmH2O; and (4) other: (i) absence of unstable limb and spinal fractures, (ii) absence of severe underlying hepatic/renal disease or new progressively worsening hepatic/renal impairment, (iii) absence of active hemorrhage, and (iv) temperature ≤ 38.5 C” (Zhao et al., 2020).
• **Exercise Stop Criteria:** For patients with COVID-19, delay the exercise program if fever or other signs and/or symptoms of COVID-19 infection exist. Assess exercise continuation individually. In general, patients with mild to moderate symptoms can gradually resume the exercise program after one week with no fever and 48 h with no symptoms. If possible, do not suspend cardiac rehabilitation components but provide them using telerehabilitation tools.
• For critically ill patients, it is recommended by Zhao et al. (2020) that “early rehabilitation to be discontinued immediately if the following conditions occur: (1) Respiratory system: (i) SpO2 < 90% or decrease by > 4% from baseline, (ii) respiratory rate > 40 bpm, (iii) ventilator resistance, and (iv) artificial airway dislodgement or migration; (2) cardiovascular system: (i) systolic blood pressure < 90 or > 180 mmHg, (ii) M.A.P. < 65 or > 110 mmHg, or > 20% change compared with baseline, (iii) heart rate < 40 or > 120 beats/min, and (iv) new arrhythmia and myocardial ischemia; (3) nervous system: (i) loss of consciousness and (ii) irritability; and (4) other: (i) discontinuation of any treatment or removal of monitoring tube connected to the patient; (ii) patient-perceived heart palpitations, exacerbation of dyspnea or shortness of breath, and intolerable fatigue; and (iii) falls inpatient” (Zhao et al., 2020).

### Special Consideration for Geriatric Rehabilitation

We identified eight publications that addressed rehabilitation of older adults (Alliance, 2020; Chen et al., 2020; Etard et al., 2020; Gómez-Moreno et al., 2020; Ismail, 2020; Jiménez-Pavón et al., 2020; Verduzco-Gutierrez et al., 2020; Zeng et al., 2020). Publication dates ranged from February 4 to July 6, 2020. Recommendations were from 8 countries [France (*n* = 1), Mexico (*n* = 1), Egypt (*n* = 1), United States (*n* = 3), China (*n* = 2), Spain (*n* = 1), Denmark (*n* = 1), and Canada (*n* = 1), two studies included data from multiple countries (China, Denmark, United States; Spain, United States)]. Eight institutions participated in developing these guidelines, including hospitals, scientific societies, and universities (Supplementary Table).

Of the eight publications, four were developed by researchers and three were developed by rehabilitation and medical professionals. The last publication did not report the group involved in developing the recommendations. Of the eight publications, six were developed based on expert opinion and/or clinical experience, and two were developed using evidence-based methods including systematic review, survey and observational studies.

### Summary of Key Recommendations

• Comprehensive Geriatric Assessment (C.G.A.) for frail seniors with rehabilitative needs should remain a priority. C.G.A. includes interprofessional geriatric assessment, physical assessment findings, analysis and synthesis of the clinical profile, and development of a collaborative plan and follow-up plan of care.
• Consider prehabilitation with the frail or at-risk patient. Prehabilitation includes interventions that aim to preventing or reducing physical impairments caused by physical stressors. Examples include cancer or surgical prehabilitation to improve treatment-related morbidity and mortality, and psychological health outcomes.
• Recognize that caregivers are essential to the care of frail seniors and are key in many settings to the provision of care. Caregivers often serve as a liaison between patients and clinicians and are involved in day-to-day decision-making and care delivery. They should therefore be included in the healthcare teams’ communication and care planning. Caregivers should be given access to necessary resources. In the context of the current pandemic, caregivers will require personal protective equipment (P.P.E.) with instruction in proper donning and doffing techniques.
• Exercise frequency: The international guidelines of physical activity for older people recommend five days per week. At the time of the pandemic, which is associated with quarantine or lockdown, the exercise frequency should increase to 5–7 days per week with adaptation in volume and intensity (Jiménez-Pavón et al., 2020).
• Exercise volume: The guidelines recommend at least 150–300 min per week of aerobic exercise and two resistance training sessions per week. Under restriction of movement due to the COVID-19 pandemic, it should increase to 200–400 min per week across 5–7 days to offset the lower levels of daily physical activity. A minimum of 2–3 days per week of resistance exercise should be recommended. Mobility training, balance and coordination exercises should be performed on all the training days (Jiménez-Pavón et al., 2020).
• Exercise intensity: The guidelines suggest moderate intensity for most of the sessions and some amount of vigorous exercise weekly. Because vigorous-intensity exercise may inhibit the immune system, especially in sedentary people, moderate-intensity (40–60% heart rate reserve or 65–75% of maximal heart rate) should be recommended for older people during restriction of movement due to COVID-19 pandemic (Jiménez-Pavón et al., 2020).
• A number of measures have been recommended to reduce the risk of COVID-19 outbreak in institutions caring for older adults:
  ◦ Lockdowns, suspension of visits and personal aids, secured supply chains, isolation of cases, extended barrier measures, sanitation, limitation of internal activities, etc.
  ◦ Public information and communication campaigns should be directed to protect older adults, make them noticeable, and offer strong psychological support to the nursing staff.
  ◦ Reinforce the communication between nursing staff and families at the end of a resident’s life and after death.
  ◦ A palliative approach of care should be proposed within the impacted institutions after accounting for ethical considerations to decrease the burden on general hospitals.
• Physiatrist outpatient in-person visits can be transitioned to virtual physical exams (telemedicine), which can be delivered using virtual workflow (before, during, and after the visit) during natural disasters such as the current pandemic due to COVID-19.

## Discussion

In this review, we pooled rehabilitation recommendations for various settings, including acute care, critical care, and different post-acute settings. Recommendations for education interventions and special consideration for older adult rehabilitation were also presented. Most of the recommendations were based on expert opinions and/or consensus. Based on the GRADE approach, the overall quality of the recommendations was deemed fair, and most of the individual recommendations were graded as strong.

Overall, the evidence suggests that continuity of rehabilitation services is critically important to maintain function and participation among patients during the COVID-19 pandemic. Yet, many barriers exist across healthcare settings. Globally, healthcare systems have needed to rapidly adapt to the different waves of the current pandemic. Telehealth has been one of the areas with higher development, emerging as a good approach to keep many aspects of healthcare, including rehabilitation, running during this time. Technology has been a key tool to help provide this continuity of healthcare attention to our patients and will likely continue having a significant role in the future. Even though the role of telerehabilitation is not yet fully understood, it is definitely “here to stay” as part of our future healthcare practice. Our guidelines acknowledge this change and include telerehabilitation recommendations and strategies to help rehabilitation professionals deliver care.

The recommendations for critical care/I.C.U. and acute settings address the need for a multidisciplinary care program in patients with COVID-19 pneumonia or acute respiratory distress syndrome (ARDS), including pulmonary rehabilitation, physical and neuropsychological recovery, and nutritional support provided early on and tailored to the unique needs of each patient. The recommendations for post-acute rehabilitation address the importance of individual assessment of neurorehabilitation, speech, musculoskeletal, respiratory, and cardiac rehabilitation following the specific protocols provided. Education of patients, caregivers and families is at the center of disease management. Specific criteria to start or stop a rehabilitation services are also provided, as are recommendations for frail seniors. The recommendations can be used to guide rehabilitation professionals in their decision-making and patient management.

We acknowledge the limitations of this review which include lack of higher levels of evidence among primary studies [e.g., randomized controlled trials (R.C.T.s)]. Although systematic reviews and meta-analyses of R.C.T.s and/or observational studies provide a more measured approach for efficacy of a treatment, we did not include them in our review as we did not find enough primary studies to conduct a systematic review on COVID-19 rehabilitation recommendations. Most publications in our review were expert opinion and clinical recommendations without systematic reviews. We synthesized and summarized the eligible publications with the best available recommendations.

The COVID-19 pandemic and the evidence addressing it are rapidly evolving. The recommendations included in this article will be updated in the near future to incorporate the most recent evidence. The COVID-19 vaccine may decrease the impact of COVID-19 and might modify some of the approaches proposed in this document, as they reflect evidence published before vaccine introduction.

In conclusion, we have combined the latest research findings and expert opinions to develop acute and post-acute rehabilitation recommendations. Further ongoing updates are warranted in order to incorporate the emerging evidence into rehabilitation guidelines.

## Author Contributions

All authors were involved in critical revision and approval of the manuscript’s design, conception, and analysis.

## Conflict of Interest

YC was employed by OrthoEvidence Inc. The remaining authors declare that the research was conducted in the absence of any commercial or financial relationships that could be construed as a potential conflict of interest.

## Publisher’s Note

All claims expressed in this article are solely those of the authors and do not necessarily represent those of their affiliated organizations, or those of the publisher, the editors and the reviewers. Any product that may be evaluated in this article, or claim that may be made by its manufacturer, is not guaranteed or endorsed by the publisher.
