# Supplementary material for: Rehabilitation at the Time of Pandemic: Patient Journey Recommendations
Source: Front Aging Neurosci. 2022 Apr 12;14:781226. doi: 10.3389/fnagi.2022.781226 (PMC9042427; doi:10.3389/fnagi.2022.781226)
Supplement: Supplementary file 1 [file Data_Sheet_1.docx]

1. **SEARCH STRATEGY**

**Date: August 1, 2020**

1. **MEDLINE = 509**

1. exp Rehabilitation/ or exp Allied Health Personnel/ or exp Allied Health Occupations/ or (rehab* or physio or physiotherap* or physical therap* or allied health or PT).mp.

2. (Rehabilitation or Allied Health Personnel or Allied Health Occupation* or rehab* or physio or physiotherap* or physical therap* or allied health or PT).mp. [mp=title, abstract, original title, name of substance word, subject heading word, floating sub-heading word, keyword heading word, organism supplementary concept word, protocol supplementary concept word, rare disease supplementary concept word, unique identifier, synonyms]

3. exp Coronavirus/ or exp Coronavirus Infections/ or coronaviru*.mp. or "corona virus*".mp. or ncov*.mp. or n-cov*.mp. or "novel cov".mp. or COVID-19.mp. or COVID19.mp. or COVID-2019.mp. or COVID2019.mp. or SARS-CoV-2.mp. or SARSCoV-2.mp. or SARSCoV2.mp. or SARSCoV19.mp. or SARS-Cov-19.mp. or SARSCov-19.mp. or SARSCoV2019.mp. or SARS-Cov-2019.mp. or SARSCov-2019.mp. or "severe acute respiratory syndrome coronaviru*".mp. or "severe acute respiratory syndrome cov 2".mp. or "2019 ncov".mp. or 2019ncov.mp.

4. 1 or 2

5. 3 and 4

1. **PUBMED = 511**

**Pneumonia, Viral/or Coronavirus Infection or Betacoronavirus**

- "coronavirus"[MeSH Terms] OR "coronavirus infections"[MeSH Terms] OR "coronaviru*"[Title/Abstract] OR "corona virus"[Title/Abstract] OR "ncov*"[Title/Abstract] OR "n cov*"[Title/Abstract] OR "novel cov"[Title/Abstract] OR "COVID-19"[Title/Abstract] OR "COVID19"[Title/Abstract] OR "COVID-2019"[Title/Abstract] OR "COVID2019"[Title/Abstract] OR "SARS-CoV-2"[Title/Abstract] OR "SARSCoV-2"[Title/Abstract] OR "sarscov2"[Title/Abstract] OR "SARSCoV19"[Title/Abstract] OR "sars cov 19"[Title/Abstract] OR "severe acute respiratory syndrome cov2"[Title/Abstract] OR "2019 ncov"[Title/Abstract] OR "2019ncov"[Title/Abstract] OR "severe acute respiratory disease"[Title/Abstract]

**Rehabilitation**

- "rehabilitation"[MeSH Terms] OR "allied health personnel"[MeSH Terms] OR "allied health occupations"[MeSH Terms] OR "rehab*"[Title/Abstract] OR "physio"[Title/Abstract] OR "physiotherap*"[Title/Abstract] OR "physical therap*"[Title/Abstract] OR "allied health"[Title/Abstract] OR "PT"[Title/Abstract]

1. **CINAHL = 177**

"coronavirus" OR "coronavirus infections" OR "coronaviru*" OR "corona virus" OR "ncov*"[Title/Abstract] OR "n cov*"[Title/Abstract] OR "novel cov"[Title/Abstract] OR "COVID-19"[Title/Abstract] OR "COVID19"[Title/Abstract] OR "COVID-2019" OR "COVID2019" OR "SARS-CoV-2" OR "SARSCoV-2" OR "sarscov2" OR "SARSCoV19" OR "sars cov 19" OR "severe acute respiratory syndrome cov2" OR "2019 ncov" OR "2019ncov" OR "severe acute respiratory disease"

rehabilitation or "allied health personnel" or "allied health occupations" or "rehab*" or "physio" or "physiotherap*" or "physical therap*" or "allied health" or "PT" or "OT"

1. **Embase = 2022**

1. exp Rehabilitation/ or exp Allied Health Personnel/ or exp Allied Health Occupations/ or (rehab* or physio or physiotherap* or physical therap* or allied health or PT).mp.

2. (Rehabilitation or Allied Health Personnel or Allied Health Occupation* or rehab* or physio or physiotherap* or physical therap* or allied health or PT).mp. [mp=title, abstract, original title, name of substance word, subject heading word, floating sub-heading word, keyword heading word, organism supplementary concept word, protocol supplementary concept word, rare disease supplementary concept word, unique identifier, synonyms]

3. exp Coronavirus/ or exp Coronavirus Infections/ or coronaviru*.mp. or "corona virus*".mp. or ncov*.mp. or n-cov*.mp. or "novel cov".mp. or COVID-19.mp. or COVID19.mp. or COVID-2019.mp. or COVID2019.mp. or SARS-CoV-2.mp. or SARSCoV-2.mp. or SARSCoV2.mp. or SARSCoV19.mp. or SARS-Cov-19.mp. or SARSCov-19.mp. or SARSCoV2019.mp. or SARS-Cov-2019.mp. or SARSCov-2019.mp. or "severe acute respiratory syndrome coronaviru*".mp. or "severe acute respiratory syndrome cov 2".mp. or "2019 ncov".mp. or 2019ncov.mp.

4. 1 or 2

5. 3 and 4

1. **List of the included studies**

1. Hosey MM, Needham DM. Survivorship after COVID-19 ICU stay. Nat Rev Dis Primers. 2020;6(1):60.

2. Masiero S, Zampieri D, Del Felice A. The Place of Early Rehabilitation in Intensive Care Unit for COVID-19. Am J Phys Med Rehabil. 2020;99(8):677-8.

3. Nakamura K, Nakano H, Naraba H, Mochizuki M, Hashimoto H. Early rehabilitation with dedicated use of belt-type electrical muscle stimulation for severe COVID-19 patients. Crit Care. 2020;24(1):342.

4. Amatya B, Khan F. Rehabilitation Response in Pandemics. Am J Phys Med Rehabil. 2020;99(8):663-8.

5. Yu P, Wei Q, He C. Early Rehabilitation for Critically Ill Patients With COVID-19: More Benefits Than Risks. Am J Phys Med Rehabil. 2020;99(6):468-9.

6. Kurtaiş Aytür Y, Füsun Köseoglu B, Özyemişci Taşkıran Ö, Kutay Ordu Gökkaya N, Ünsal Delialioğlu S, Sonel Tur B, et al. Pulmonary rehabilitation principles in SARS-COV-2 infection (COVID-19): The revised guideline for the acute, subacute, and post-COVID-19 rehabilitation. Turk J Phys Med Rehabil. 2021;67(2):129-45.

7. Pinto TF, Carvalho CRF. SARS CoV-2 (COVID-19): lessons to be learned by Brazilian Physical Therapists. Braz J Phys Ther. 2020;24(3):185-6.

8. Qu JM, Wang C, Cao B, Physicians CTSaCAoC. Guidance for the management of adult patients with coronavirus disease 2019. Chin Med J (Engl). 2020;133(13):1575-94.

9. Sheehy LM. Considerations for Postacute Rehabilitation for Survivors of COVID-19. JMIR Public Health Surveill. 2020;6(2):e19462.

10. Recommendations AHSSAGC-. Rapid Evidence Report. 2020.

11. Thomas P, Baldwin C, Bissett B, Boden I, Gosselink R, Granger CL, et al. Physiotherapy management for COVID-19 in the acute hospital setting: clinical practice recommendations. J Physiother. 2020;66(2):73-82.

12. Medicine CAoR, Medicine RRCoCAoR, Rehabilitation CRGoCSoPMa. [Recommendations for respiratory rehabilitation of coronavirus disease 2019 in adult]. Zhonghua Jie He He Hu Xi Za Zhi. 2020;43(4):308-14.

13. Vitacca M, Carone M, Clini EM, Paneroni M, Lazzeri M, Lanza A, et al. Joint Statement on the Role of Respiratory Rehabilitation in the COVID-19 Crisis: The Italian Position Paper. Respiration. 2020;99(6):493-9.

14. Sañudo B, Seixas A, Gloeckl R, Rittweger J, Rawer R, Taiar R, et al. Potential Application of Whole Body Vibration Exercise For Improving The Clinical Conditions of COVID-19 Infected Individuals: A Narrative Review From the World Association of Vibration Exercise Experts (WAVex) Panel. Int J Environ Res Public Health. 2020;17(10).

15. Kalirathinam D, Guruchandran R, Subramani P. Comprehensive physiotherapy management in covid-19 – a narrative review. Scientia Medica. 2020;30:1-9.

16. Arenivas A, Carter KR, Harik LM, Hays KM. COVID-19 neuropsychological factors and considerations within the acute physical medicine and rehabilitation setting. Brain Inj. 2020;34(8):1136-7.

17. Handu D, Moloney L, Rozga M, Cheng FW. Malnutrition Care During the COVID-19 Pandemic: Considerations for Registered Dietitian Nutritionists. J Acad Nutr Diet. 2021;121(5):979-87.

18. Boldrini P, Bernetti A, Fiore P, SIMFER Executive Committee SIMFCfIA. Impact of COVID-19 outbreak on rehabilitation services and Physical and Rehabilitation Medicine physicians' activities in Italy. An official document of the Italian PRM Society (SIMFER). Eur J Phys Rehabil Med. 2020;56(3):316-8.

19. Bartolo M, Intiso D, Lentino C, Sandrini G, Paolucci S, Zampolini M, et al. Urgent Measures for the Containment of the Coronavirus (Covid-19) Epidemic in the Neurorehabilitation/Rehabilitation Departments in the Phase of Maximum Expansion of the Epidemic. Front Neurol. 2020;11:423.

20. Pandian JD, Sebastian I. Management of Acute Stroke During COVID-19 Global Pandemic. Journal of Stroke Medicine 2020;3:7-9.

21. Wade DT. Rehabilitation after COVID-19: an evidence-based approach. Clin Med (Lond). 2020;20(4):359-65.

22. Kho ME, Brooks, D., Namasivayam-MacDonald, A., Sangrar, R. and Vrkljan, B. Rehabilitation for Patients with COVID-19. Guidance for Occupational Therapists, Physical Therapists, Speech-Language Pathologists and Assistants. School of Rehabilitation Science, McMaster University; 2020.

23. Brugliera L, Spina A, Castellazzi P, Cimino P, Arcuri P, Deriu MG, et al. Rehabilitative of COVID-19 patients with acute lower extremity Ischemia and amputation. J Rehabil Med. 2020;52(9):jrm00094.

24. Yu HP, Jones AY, Dean E, Liisa Laakso E. Ultra-shortwave diathermy - a new purported treatment for management of patients with COVID-19. Physiother Theory Pract. 2020;36(5):559-63.

25. Bij de Vaate E, Gerrits KHL, Goossens PH. Personalized recovery of severe COVID19: Rehabilitation from the perspective of patient needs. Eur J Clin Invest. 2020;50(7):e13325.

26. Barker-Davies RM, O'Sullivan O, Senaratne KPP, Baker P, Cranley M, Dharm-Datta S, et al. The Stanford Hall consensus statement for post-COVID-19 rehabilitation. Br J Sports Med. 2020;54(16):949-59.

27. Gómez-Moreno C, Hernández-Ruiz V, Hernández-Gilsoul T, Avila-Funes JA. CLINICAL DECISION MAKING IN OLDER ADULTS WITH COVID-19 IN DEVELOPING COUNTRIES: LOOKING BEYOND CHRONOLOGICAL AGE. Rev Invest Clin. 2020;72(3):127-34.

28. Mammi P, Ranza E, Petraglia F, Rampello A, Pessina F, Zaccaria B, et al. In Response to: Simpson and Robinson: Rehabilitation After Critical Illness in People With COVID-19 Infection. Am J Phys Med Rehabil. 2020;99(8):679-80.

29. Piepoli MF. Editor's presentation: Staying healthy and fighting cardiovascular disease at the time of COVID. Eur J Prev Cardiol. 2020;27(9):899-902.

30. Schmidt C, Magalhães S, Barreira A, Ribeiro F, Fernandes P, Santos M. Cardiac rehabilitation programs for heart failure patients in the time of COVID-19. Rev Port Cardiol (Engl Ed). 2020;39(7):365-6.

31. Zhao HM, Xie YX, Wang C, Medicine CAoR, Medicine RRCoCAoR, Rehabilitation CRGoCSoPMa. Recommendations for respiratory rehabilitation in adults with coronavirus disease 2019. Chin Med J (Engl). 2020;133(13):1595-602.

32. Yang LL, Yang T. Pulmonary rehabilitation for patients with coronavirus disease 2019 (COVID-19). Chronic Dis Transl Med. 2020;6(2):79-86.

33. Jangra MK, Saxena A. Significance of physiotherapy in "SARS-CoV-2/COVID-19: An Epidemic". Ann Thorac Med. 2020;15(3):179-80.

34. Polastri M, Nava S, Clini E, Vitacca M, Gosselink R. COVID-19 and pulmonary rehabilitation: preparing for phase three. Eur Respir J. 2020;55(6).

35. Galiuto L, Crea F. Let Us Not Forget Cardiovascular Diseases During COVID-19 Pandemic: the Role of Cardiac Prevention and Rehabilitation. SN Compr Clin Med. 2020:1-4.

36. Jalali M, Shahabi S, Lankarani KB, Kamal M, Mojgani P. COVID-19 and disabled people: perspectives from Iran. Disability & Society. 2020;35:844-7.

37. Griffin MF. An Invited Commentary on: Emergency and essential surgical healthcare services during COVID-19 in low- and middle-income countries: A perspective. Int J Surg. 2020;79:265-6.

38. Kemps HMC, Brouwers RWM, Cramer MJ, Jorstad HT, de Kluiver EP, Kraaijenhagen RA, et al. Recommendations on how to provide cardiac rehabilitation services during the COVID-19 pandemic. Neth Heart J. 2020;28(7-8):387-90.

39. Aytür YK, Köseoğlu BF, Taşkıran ÖÖ, Ordu-Gökkaya NK, Delialioğlu SÜ, Tur BS, et al. Pulmonary rehabilitation principles in SARS-COV-2 infection (COVID-19): A guideline for the acute and subacute rehabilitation. Turkish Journal of Physical Medicine & Rehabilitation (2587-0823). 2020;66(2):104-20.

40. Alliance RC. Frail Senior: Guidance on Best Practice Rehabilitative Care in the Context of COVID-19. 2020.

41. Verduzco-Gutierrez M, Bean AC, Tenforde AS, Tapia RN, Silver JK. How to Conduct an Outpatient Telemedicine Rehabilitation or Prehabilitation Visit. PM R. 2020;12(7):714-20.

42. Chen P, Mao L, Nassis GP, Harmer P, Ainsworth BE, Li F. Coronavirus disease (COVID-19): The need to maintain regular physical activity while taking precautions. J Sport Health Sci. 2020;9(2):103-4.

43. Ismail AA. Cancelled elderly exercise sessions during the COVID-19 crisis: can physical therapists help from their homes? European Journal of Physiotherapy. 2020;22:235 -

44. Jiménez-Pavón D, Carbonell-Baeza A, Lavie CJ. Physical exercise as therapy to fight against the mental and physical consequences of COVID-19 quarantine: Special focus in older people. Prog Cardiovasc Dis. 2020;63(3):386-8.

45. Etard JF, Vanhems P, Atlani-Duault L, Ecochard R. Potential lethal outbreak of coronavirus disease (COVID-19) among the elderly in retirement homes and long-term facilities, France, March 2020. Euro Surveill. 2020;25(15).

46. Zeng B, Chen D, Qiu Z, Zhang M, Wang G, Wang J, et al. Expert consensus on protocol of rehabilitation for COVID-19 patients using framework and approaches of WHO International Family Classifications. Aging Med (Milton). 2020;3(2):82-94.

**Supplementary Table**

| **Patient Journey Recommendations** | | **Direction & Strength of Recommendation** |  |
| --- | --- | --- | --- |
| **No.** | **Acute/ICU Rehabilitation** |  |  |
| 1 | In patients with COVID-19 pneumonia or acute respiratory distress syndrome (ARDS), include a multidisciplinary/holistic care program on pulmonary rehabilitation tailored to the unique needs of each patient by giving meticulous attention to the delivery of evidence-based critical care interventions, early and sustained comprehensive rehabilitation targeting physical and neuropsychological recovery, and by evaluation of adequate social support. It is recommended for physiotherapy to begin in the acute inpatient setting and to continue after transfer to inpatient rehabilitation. | Strong For |  |
| 2 | Optimal critical interventions of ARDS include strategies such as lung-protective mechanical ventilation and prone positioning in addition to evidence-based strategies for evaluation and management of pain, sedation, delirium, immobility, and sleep. | Strong For |  |
| 3 | The potential benefits of rehabilitation for the COVID-19 patient treated in an acute care setting must also be weighed against the risk of viral transmission to rehabilitation health care providers. | Strong For |  |
| 4 | Beginning early rehabilitation in the intensive care unit (ICU) to promote rapid functional recovery given some patients may present with low exercise capacity, low physical activity level, increased sedentary behavior, dyspnea on exertion, and poorer quality of life after hospital discharge. | Strong For |  |
| 5 | The suggested airway clearance techniques include positioning, active cycle of breathing, manual and ventilator hyperinflation, percussion and vibrations, positive expiratory pressure therapy (PEP), assisted or stimulated cough manoeuvres, airway suctioning, and mechanical insufflation-exsufflation. | Weak For |  |
| 6 | Respiratory management of severe cough and increase in breathing include lung recruitment and sputum expulsion and do not require therapists to have long periods of patient contact. The recommended treatment methods include high-frequency chest wall oscillation and oscillatory positive expiratory pressure (OPEP). | Weak For |  |
| 7 | Positioning management includes gradually changing positioning in more vertical anti-gravity postures when physiological status permits. For example, raising the head of the bed by increasing degrees until the patient can maintain an upright position followed by placing the lower edge of one pillow on one-third scapula in the supine position to prevent head hyperextension and by placing another pillow below the popliteal fossa to relax the lower limbs and abdomen. It is recommended this positioning management be carried out in 30-min sessions for three sessions per day. Prone position ventilation may also be carried out in patients with ARDS for 12 plus hours. | Strong For |  |
| 8 | Ensuring the safety of pipelines, circuits, and normal running of equipment, and prevention of adverse events such as falls during rehabilitation in the acute care hospital. | Strong For |  |
| 9 | Ensure safety and integrity of tubing and lines to prevent inadvertent disconnection or detachment during mobilization and rehabilitation interventions.  Monitor vital signs during rehabilitation sessions and vital sign responses to rehabilitation interventions.  Consider intensity and duration of rehabilitation activities and sessions and adjust accordingly for patients with poor physical status.  Ensure lower intensity and shorter duration activities, exercises, and sessions for patients with poor physical status.  Ensuring rehabilitation sessions do not exacerbate fatigue by limiting it to 30 minutes.  To maintain or improve joint integrity, range of motion, and muscle strength, exercise considerations include passive, active-assisted, those with active range of motion or those that are resistance based.  Considerations for mobility/mobilization/exercises include active range of motion (ROM) exercises through the full available range. Examples include exercises that promote side-to-side position changes, bed mobility, sitting at the edge of the bed, moving from the bed to chair, sitting in a chair, standing, stepping in place, walking, tilt table, standing hoists, upper/lower cycle ergometry, and exercise programs.  For patients receiving sedatives or patients with loss of consciousness, cognitive dysfunction, or limited physiological capacity, exercises include in-bed cycling, passive ROM exercises, stretching exercises, and neuromuscular electrical stimulation. | Strong For |  |
| 10 | It is recommended physiotherapists and other healthcare professionals with similar training plans provide rehabilitation interventions to address musculoskeletal, neurological, and cardiopulmonary system dysfunction in patients with comorbidities, with significant functional decline, or at risk of ICU-acquired weakness. | Strong For |  |
| 11 | When appropriate, consider including respiratory muscle training and peripheral muscle strength training as an exercise regimen. | Strong For |  |
| 12 | The Whole-Body Vibration (WBV) benefits for managing individuals with COVID-19 include: (a) reduction in fatigue and reduced risk of dyspnea, (b) improvements in inflammatory status and cellular homeostasis, and (c) improvement in quality of life for bed-bound and ICU-bound patients. | Strong For |  |
| 13 | Use Medical Research Council scale or dynamometer for manual muscle testing to evaluate peripheral muscle strength. Assess balance function right away for patients with prolonged periods of immobility. | Strong For |  |
| 14 | Reconditioning interventions are suggested for weaned patients and those with prolonged weaning from mechanical ventilation and oxygen use to improve physical function and capacity and to address motor and cognitive effects of prolonged immobilization in ICU. | Strong For |  |
| 15 | Neuropsychologists in acute rehabilitation settings are recommended to assess and treat varied cognitive presentations routinely and to be flexible in adapting cognitive evaluation methods according to the patient's functional level.  Use evidence-based interventions to promote mental health and coping skills. Count on neuropsychologists as an emotional support source on a frequent basis.  Neuropsychologists may serve a crucial role in increasing access/connectedness to natural supports outside the hospital via virtual or other safe methods. They may involve interdisciplinary collaboration and coordination among therapy staff, nursing, and family/caregivers.  Neuropsychologists can be instrumental in facilitating referrals and resources for mental health continuity of care post-discharge when behavioral health services are limited by the COVID-19 pandemic. | Strong For |  |
| 16 | For prevention of venous thrombosis of the lower limbs, use compression socks and ankle pump exercises. | Strong For |  |
| 17 | Work with the multidisciplinary team and registered dieticians (RD) to ensure adequate energy and protein intake in individuals with suspected or confirmed COVID-19 infection in the ICU who are not mechanically ventilated.  Enteral nutrition (EN) is the preferred feeding route when needs cannot be met orally. Initiate parenteral nutrition (PN) in a timely manner to treat and prevent further malnutrition if EN is not appropriate or tolerated. | Strong For |  |
| 18 | Provide critically ill patients in the ICU with frequent small feedings, including high-energy and high-protein foods and oral nutrition supplements. Initiate nutrition support if protein and energy needs cannot be met with oral intake. Consider parenteral nutrition (PN) when airway complications can occur in patients with NIV. | Strong For |  |
|  | **Post-acute Rehabilitation** |  |  |
| 19 | Inpatient rehabilitation settings: Ensure adequate delivery of interventions and development of individual rehabilitation plans for patients directly admitted from the acute care wards, including patients recovering from COVID-19 with disabling sequelae. | Strong For |  |
| 20 | Evaluate every condition for patients admitted to rehabilitation inpatient units from home or other community facilities taking into account the balance between benefits and harms, postponing the admission, and seeking other alternative options of care whenever possible. | Strong For |  |
| 21 | Assess basic cognitive and communication functions as well as dysphagia as part of Speech-Language Pathology. | Weak For |  |
| 21 | The role of occupational Therapist or healthcare professionals with similar training includes:  - Prevention, detection, and monitoring of delirium  - Assessment and management of impairments in physical and cognitive functioning  - Assessment of emotional coping strategies for patients  - Addressing mental health and psychosocial needs of patients and/or caregivers | Strong For |  |
|  |  |  |  |
| 22 | Manage all patients who are not COVID-19 suspected or confirmed cases in a designated stroke unit as per protocols.  Manage suspected or positive COVID-19 stroke patients who may be receiving suboptimal stroke care in COVID-19 designated areas till reports of testing are confirmed.  Train COVID-19 area nurses and other allied health care personnel in basic components of stroke care (e.g., blood pressure, fever and sugar monitoring, deep vein thrombosis prophylaxis, and swallowing assessment). | Weak For |  |
|  |  |  |  |
|  |  |  |  |
|  |  |  |  |
| 22 | Use self-assessment scales to identify the type of psychological dysfunction rapidly. If necessary, advise patients to visit with psychologists or ask for help through a mental health hotline. | Strong For |  |
| 23 | Allow Neurorehabilitation/neurological Units not affected or suspected by COVID19 located in multidisciplinary hospitals to admit patients with sub-acute neurological impairments due to severe acquired brain lesions or stroke coming from an acute unit of the same hospital (i.e., ICU, neurosurgery, neurology). | Weak For |  |
|  |  |  |  |
| 24 | Treat patients suspected or affected by COVID19 with neurological impairments requiring rehabilitation according to clinical conditions (hemodynamic parameters, breathing capacity, consciousness) in the room (with appropriate personal protective equipment (PPE) while maintaining a distance of <1.5m). | Strong For |  |
| 25 | Admit patients with neurological disorders requiring rehabilitation suggestive of COVID-19 infection from acute units outside hospitals without fever and cough when throat and nasal swabs are negative and after proper time assessment. | Weak For |  |
| 26 | Admit patients with neurorehabilitation/rehabilitation units located outside general and multidisciplinary hospitals with sub-acute neurological disorders negative for COVID-19 infections in order to facilitate prompt availability of intensive care units. | Weak For |  |
| 27 | Provide task-specific training to COVID-19 patients with neurological and musculoskeletal damage. | Strong For |  |
| 28 | Provide psychosocial support (using techniques such as cognitive behavioural therapy and motivational interviewing) to manage emotional disturbance, changes in self-esteem and self-confidence, and similar constructs. | Strong For |  |
| 29 | Provide psychological interventions using medical manuals and related guidelines, audio, and video for psychological counselling. | Strong For |  |
| 30 | Assess nutritional status early with consequent addition of oral food supplements; when not tolerated, transition to artificial nutrition. | Strong For |  |
|  |  |  |  |
| 31 | Use the Malnutrition Screening Tool, a quick and easy-to-use validated tool based on two questions addressing decreased intake due to poor appetite and recent unintentional weight loss for adults with COVID-19. | Strong For |  |
|  |  |  |  |
| 32 | Include in the role of Speech-Language Pathology  - Assessment and management of dysphagia post-extubation, upon decompensation and respiratory compromise  - Assessment of basic cognitive and communication functions  -Assessment and treatment of voice impairments resulting from prolonged intubation and/or to address respiratory strength and coordination. | Strong For |  |
|  |  |  |  |
|  |  |  |  |
| 33 | In the context of the COVID-19 pandemic, touching or gently palpating the patient to determine muscle and fat store losses might not be possible. In these cases, have the RD conduct a visual inspection to note indentions and bony prominences, which may be indicative of somatic losses. | Strong For |  |
| 34 | When appropriate, ensure RDs screen for food insecurity, provide guidance and resources for eating healthfully on a budget, and provide resources to improve access to healthy foods. | Strong For |  |
| 35 | Provide early nutritional supplement protocol for non-critical COVID-19 patients with severe inflammatory status and anorexia with strong reductions in food intake. | Weak For |  |
| 36 | Provide rapid intravenous administration of whey proteins, vitamins, and minerals (and cholecalciferol if insufficient) in line with dietary allowance. | Weak For |  |
| 37 | Provide personalized swallowing of rehabilitation targeting the recovery of normal feeding during the hospitalization in order to reduce the duration of admissions. | Weak For |  |
| 38 | Have RD's advice counsel and advise patients and families with suspected or confirmed COVID-19 infections who are in their homes or the outpatient setting, and on the following considerations:  -Ensure adequate intake of energy and protein by meeting, at minimum, 100% of the recommended dietary allowance for energy and protein based on age and sex. Note, these requirements can be increased based on the pathology of COVID-19 infection. High-calorie, high-protein meals and snacks can help prevent weight loss and maintain lean muscle mass. For example, RDs may advise eating vegetables with cream, butter, margarine, cheese sauce, olive oil, or salad dressing to increase energy intake and choose foods high in protein, such as milk, eggs, cheese, meats, fish, poultry, nuts, and beans.  - Good methods to increase calorie and protein intake if oral dietary intake is not adequate to meet needs (e.g., protein powders and meal-replacement shakes and bars) include nutrient-dense foods and beverages, including oral nutritional supplements if oral dietary intake is not adequate to meet needs (e.g., protein powders and meal-replacement shakes and bars).  - For individuals with difficulty coordinating chewing and breathing, beverages are a better option to increase energy intake compared to solid foods efficiently.  - Offer micronutrient supplements to address deficiencies and to help compensate for inadequate oral intake.  - Offer small and frequent meals and snacks to manage nausea, vomiting, and shortness of breath.  - Suggest foods that require little handling, preparation, or effort to eat.  - Ensure adequate intake of fluids to stay hydrated throughout the day and evening. If the patient is experiencing vomiting and diarrhea, advise patients to consume rehydration drinks. | Strong For |  |
|  |  |  |  |
|  |  |  |  |
|  |  |  |  |
|  |  |  |  |
|  |  |  |  |
|  |  |  |  |
|  |  |  |  |
| 39 | Considerations for the role of an occupational therapist or healthcare professionals with similar training to include the following:  - Providing bed optimization and positioning of seating using pressure relief principles (e.g., mattress).  - Assessment and management of activity of daily living (ADLs) and instrumental activities of daily living (IADL) to encourage early mobilization.  - Provision of assistive devices for ADLs, communication, seating, and mobility.  - Facilitating functional independence/autonomy and preparing patients for discharge. | Strong For |  |
|  |  |  |  |
|  |  |  |  |
|  |  |  |  |
| 40 | The role of physiotherapy or healthcare professionals with similar training may include the following:  - Assessment of exercise and functional capacity.  - Monitoring of pre-existing comorbid conditions.  - Exercise training and/or physical activity coaching. | Strong For |  |
|  |  |  |  |
|  |  |  |  |
| 41 | Considerations for rehabilitation evaluation include the following:(1) Clinical evaluation may include physical examination, imaging tests, laboratory tests, lung function tests, nutrition screening, and ultrasonography. (2) Exercise and respiratory function evaluation may include (i) evaluation of respiratory muscle strength: maximum inspiratory pressure/maximum expiratory pressure; (ii) muscle strength: manual muscle testing using the Medical Research Council scale; isokinetic muscle testing; (iii) joint ROM test; (iv) balance function evaluation using the Berg Balance Scale; (v) evaluation of aerobic exercise capacity using the6-minute walk test and cardiopulmonary exercise testing; and (vi) physical activity evaluation using the International Physical Activity Questionnaire and Physical Activity Scale for the Elderly. (3) Evaluation of ADL using the Barthel index or another equivalent instrument. | Weak For |  |
| 42 | Assess exercise capacity using the 6-minute walk test (with continuous oxygen saturation monitoring) and cardiopulmonary exercise testing. | Strong For |  |
| 43 | Measure function and disability with the International Physical Activity Questionnaire, Physical Activity Scale for the Elderly, and the Barthel Index to measure activities of daily living (ADLs). | Strong For |  |
| 44 | Consideration for rehabilitation disciplines includes focusing on evidence-based exercise programs to optimize respiratory and peripheral muscle strength. | Strong For |  |
| 45 | Base the types of exercises for the rehabilitation program on patient's preferences. | Strong For |  |
| 46 | The choice between providing the first‐line physiotherapy under the supervision of a general practitioner (GP) or an integrated treatment programme in a specialized rehabilitation clinic (if available) can be made for a COVID-19 survivor according to the level of coexisting cognitive and emotional dysfunction of the patient. | Weak For |  |
| 47 | For patients with high levels of premorbid participation and high‐level participation goals, ensure inpatient specialist rehabilitation treatment | Strong For |  |
| 48 | Provide geriatric rehabilitation or reactivation programmes for patients with pre-existing low levels of functioning, with severe interacting comorbidities that can hamper physical training or with low‐level participation goals. | Strong For |  |
| 49 | Develop a personalized rehabilitation plan based on a comprehensive assessment of the patient's respiratory function, exercise ability, ADL, anxiety, depression, cognitive abilities, and other key factors.  Considerations for the plan include aerobic training, peripheral and respiratory muscle strength training, guidance for performing ADL, and nutrition and psychological support. Additionally, remote online management may be used. | Strong For |  |
| 50 | Customize aerobic exercises according to the patient's underlying COVID-19 disease and residual dysfunction. These exercises may include walking, brisk walking, slow jogging, and swimming, beginning with low intensity before increasing progressively in intensity and duration. Carry out a total of 3 to 5 sessions per week, with each session lasting 20 to 30 minutes. Recommend intermittent exercises for patients who may be prone to fatigue.  Recommend progressive resistance training for strength training as follows 8 to 12 repetitions per set, 1 to 3 sets for each target muscle group, with 2-minute rest intervals between sets, at a frequency of 2 to 3 sessions per week for six weeks.  Recommend balance training in patients with comorbid balance disorders. | Strong For |  |
| 51 | Tailor the intensity, timing and modality to the individual patient's needs regardless of the type of intervention, particularly for those with severe/critical illness, older patients, obese patients, and those with comorbidity and other coexisting complications. | Strong For |  |
| 52 | Instruct patients to perform a low-intensity exercise at an intensity that does not induce fatigue the following day. | Strong For |  |
| 53 | Maintain the intensity of daily exercise between rest (1.0 metabolic equivalents [METs]) and light physical activity (<3.0 METs) with a duration of 15 to 45 minutes; with intermittent exercises. | Strong For |  |
| 54 | Provide early mobilization while respecting the patient's respiratory and hemodynamic states and can include frequent posture changes, bed mobility, sit-to-stand, simple bed exercises, and ADLs. | Weak For |  |
| 55 | Recommend exercise that increases demand upon the cardio-respiratory system are important for general fitness, and to combat fatigue, mental health issues, lack of confidence, and to improve performance of daily activities such as walking. | Strong For |  |
| 56 | Use neuromuscular electrical stimulation to assist with strengthening. | Weak For |  |
| 57 | Produce and distribute an information brochure with simple exercises for patients with milder forms of COVID-19 infection. | Strong For |  |
| 58 | Encourage partaking in a wide range of exercises, including video or app-guided, equipment-free aerobics or strength training, that can be performed at home. | Strong For |  |
| 59 | Ensure Home-Based Rehabilitation enforces physical distancing to limit COVID-19 spread and decrease the COVID-19 burden on inpatient services. | Strong For |  |
| 60 | Ensure for Home-Based Rehabilitation, enhanced home care services and outpatient rehabilitation are available and are able to provide a level of care on par with inpatient rehabilitation. | Strong For |  |
| 61 | For Home-Based Rehabilitation, provision of home-based care by home care staff utilizing precautions may be safer for patients who have recovered from COVID-19 and for other patients in the rehabilitation unit. | Strong For |  |
| 62 | Home-based therapy can be provided over the telephone or the internet (telerehabilitation). Both assessment and treatment may be provided, either synchronously (i.e., in real-time) or asynchronously (e.g., a pre-recorded customized exercise plan).  Consider the use of eHealth, internet and mobile technology, and television are other viable avenues for maintaining physical function and mental health during critical periods of social isolation. | Strong For |  |
| 63 | Make weekly telephone calls to review training activities, provide feedback, and engage patients' families as this weekly check-in may increase motivation to stay engaged in activities. | Strong For |  |
| 64 | Adjust the exercise program plan to the patients' home physical conditions. Help patients identify safe, alternative spaces for aerobic training according to current government/jurisdictional COVID guidelines. | Strong For |  |
| 65 | Home-based exercises using various safe, simple, and easily implementable exercises can maintain fitness levels while avoiding the airborne coronavirus and its variants. Home-based exercises can include strengthening exercises, activities for balance and control, stretching exercises, or a combination of these. | Strong For |  |
| 66 | Recommend at least 30 minutes of moderate physical activity every day and/or at least 20 minutes of vigorous physical activity every other day during a pandemic. Ideally, a combination of both intensities of physical activities is preferable in addition to practicing strengthening-type activities on a regular basis. | Strong For |  |
| 67 | For patients with COVID-19, rehabilitation aims to relieve symptoms of dyspnoea, psychological distress and to improve participation in rehabilitation, physical function and quality of life. | Strong For |  |
| 68 | Considerations for mild COVID-19 illness: (1) Exercise intensity may include Modified BORG Dyspnea Scale ≤3 points, fatigue should be absent on Day 2 preferably; (2) exercise frequency can be twice a day in15 to 45 minutes duration per session, with longer duration of 1hour after meals; and (3) type of exercises may include breathing exercise, tai chi, or square dancing. | Strong For |  |
| 69 | Considerations for Physical activity and exercise recommendations for moderate COVID-19 illness include(i) Exercise intensity, between rest (1.0 metabolic equivalents [METs]) and light exercise (<3.0 METs); (ii) Exercise frequency, twice a day, 1 hour after a meal; (iii) duration maybe 15 to 45 minutes per session depending on patient's physical status with patients prone to fatigue or are physically weak may perform intermittent exercise; (iv) type of exercise, breathing exercises, stepping, tai chi, and exercises that can prevent thrombosis. | Strong For |  |
| 70 | Consider low-intensity physical activity (≤3 METs or equivalent) initially, particularly for patients who require oxygen therapy, while concurrently monitoring vital signs (heart rate, pulse oximetry and blood pressure). Recommend any gradual increases in physical activity based on symptoms. | Strong For |  |
| 71 | Provide rehabilitation guidance for (i) basic ADLs or the patient's ability in transferring, getting dressed, toileting, and bathing are assessed; (ii) instrumental ADLs (IADLs) or the IADL of the patients to identify any issues. Carry out targeted intervention under the guidance of the occupational therapist or the health professional with similar training. | Strong For |  |
| 72 | Include for respiratory assessment evaluation of dyspnea, thoracic activity, diaphragmatic activity and amplitude, respiratory muscle strength (maximal inspiratory and expiratory pressures), respiratory pattern, and frequency. Additionally, assess cardiac status. | Strong For |  |
| 73 | Conduct evaluation and monitoring throughout the pulmonary rehabilitation program. | Strong For |  |
| 74 | Layout, the short-term goal of pulmonary rehabilitation, as to alleviate dyspnea and relieve anxiety and depression. | Weak For |  |
| 75 | Layout the long-term goal of pulmonary rehabilitation as to preserve the patient's function to the maximum extent, improve their quality of life, and facilitate their return to society. | Strong For |  |
| 76 | Physiotherapy and cardiorespiratory therapists may play a vital role in the respiratory management and rehabilitation of patients with SARS-CoV-2/COVID-19. | Strong For |  |
| 77 | In hospitalized patients with mild/moderate disease, provide respiratory rehabilitation to potentially improve symptoms (dyspnea, anxiety and depression), physical capacity and quality of life. | Strong For |  |
| 78 | Minimize operations that can increase the risk of infection, such as -ingested cough, expiration training, and tracheal compression. Use a sealed plastic bag to cover the mouth during expectoration to prevent infection. In addition, carry out pulmonary rehabilitation of patients with COVID-19 through educational videos, brochures, remote consultations, or online teaching to save protective equipment and avoid cross-infection. | Strong For |  |
| 79 | Suggest physiotherapy in patients with copious secretions that are unable to clear airways independently and adequately. | Weak For |  |
| 80 | Suggest the following primary intervention measures for respiratory rehabilitation: airway clearance, reduction of dyspnea breathing control, physical activity, and exercise. | Weak For |  |
| 81 | Airway clearance includes (i) dilation during deep breathing exercises to help sputum expectoration and (ii) using a sealed plastic bag when coughing to avoid viral transmission. | Weak For |  |
| 82 | Ensure airway remains unobstructed during rehabilitation interventions and use thoracic expansion exercises to support sputum clearance. | Strong For |  |
| 83 | Consider prone positioning, lung-protective ventilation, and extracorporeal membrane oxygenation for refractory hypoxemia for the management of severe cases of airway clearance. | Weak For |  |
| 84 | The physiotherapist can use postural drainage and various manual/mechanical airway clearance techniques to clear the airways. | Weak For |  |
| 85 | Use inspiratory muscle training and breathing exercises to improve dyspnea. | Weak For |  |
| 86 | Considerations for breathing control include: (i) for positioning adopt an upright sitting position; with a leaning forward position for patients with shortness of breath; (ii) for maneuvers have patients use the accessory muscles of the shoulders and neck to relax and to inhale through the nose slowly and to slowly exhale through the mouth while paying attention to expand the lower chest. | Strong For |  |
| 87 | Include inspiratory muscle training in the post-acute phase if inspiratory muscles are weak. Consider adding deep, slow breathing, thoracic expansion (with shoulder elevation), diaphragmatic breathing, mobilization of respiratory muscles, airway clearance techniques (as needed), and positive expiratory pressure devices based on assessed needs | Weak For |  |
| 88 | Post-discharge considerations from acute care include two sessions of 10 minutes of respiratory rehabilitation per week for six weeks for improvements in respiratory function, endurance, quality of life, and depression. | Strong For |  |
| 89 | Rehabilitation considerations post-discharge include respiratory muscle training with a positive expiratory pressure device, cough exercises, diaphragmatic training (using 1 to 3 kilograms of weight on the abdomen in supine), chest stretching, and pursed-lip breathing. | Weak For |  |
| 90 | Other considerations post-discharge includes Chinese medicine techniques such as tai chi, the Qigong 6-character mnemonic, guided breathing, and Baduanj in qigong. | Weak For |  |
| 91 | Consider cardiac and neurological involvements secondary to COVID-19when planning the pulmonary rehabilitation program. | Strong For |  |
| 92 | Recommendations for respiratory training include arranging respiratory mode training in combination with the evaluation results, such as body position management, adjustment of respiratory rhythm, traction of respiratory muscle group breathing exercise, and expectoration training if the patient has symptoms, such as shortness of breath, wheezing, and difficulty in expectoration after discharge. | Weak For |  |
| 93 | For breathing exercises, use the evaluation results to arrange the intentional breathing exercise and airway clearance technique if shortness of breath, wheezing, and difficulty in expelling sputum occur in patients after discharge. Considerations for breathing exercises include posture management, adjustment of breathing rhythm, thoracic expansion training, and mobilization of respiratory muscle groups.  Use forced airway clearance techniques at the early stages of airway clearance after discharge in patients with chronic airway disease to expel sputum and reduce coughing and energy consumption, followed by positive expiratory pressure/OPEP as aids. | Weak For |  |
| 94 | To define a rehabilitation programme for post-COVID-19 patients, mirroring the algorithm of pulmonary rehabilitation for patients with chronic respiratory conditions is a widely accepted option that is evidence-based and well recognised. | Strong For |  |
| 95 | Provide respiratory rehabilitation guidance for patients in quarantine through audio, video, brochures, or remote consultations. | Strong For |  |
| 96 | Give cardiovascular protection during COVID-19 infection along with adequate cardiac rehabilitation programs to survivors of the disease. | Strong For |  |
| 97 | Ensure home-based cardiac rehabilitation (CR) programs consist of the same core components as center-based programs. | Strong For |  |
| 98 | Most programs are hybrid programs as they may include a number of sessions during the first weeks in which patients are assessed and monitored during exercise to teach patients how to self-monitor exercise intensity and recognize safety alert signals. | Strong For |  |
| 99 | Telemonitoring during training sessions can include varying degrees of technology-assisted assessment ranging from a simple format using only a logbook and structured telephone calls to wearable sensors, such as heart rate monitors, accelerometers or pedometers, or a high-tech approach such as remote ECG telemetry monitoring or synchronous videoconferencing.  Decisions on what approach to use and the degree of technological sophistication required may depend on patient-related factors, including cardiovascular risk, digital skills and personal preferences, and provider-related factors such as logistical conditions that include staff training and availability of technological equipment.  The use of videoconferencing technologies, which enable patients to interact with the cardiac rehabilitation team (and potentially with other patients), currently appears to be a useful way to lessen the mental and physical consequences of social isolation imposed by the COVID-19 pandemic. | Strong For |  |
| 100 | Given possible, although minor, myocardial damage in COVID-19 survivors, cardiac evaluation of all patients with previously known or suspected cardiovascular disease is desirable. Furthermore, in order to prescribe a personalized exercise program to optimize secondary prevention, a cardiovascular and respiratory functional evaluation is suggested with a cardiopulmonary stress test is suggested. | Weak For |  |
| 101 | For safety concerns of patients and their families, the use of a hybrid approach with supervised sessions in the cardiac rehabilitation unit is recommended with an initial start of low-intensity exercise prescription in order to promote patients' confidence and adherence, followed by weekly telephone calls to discuss exercise progression and any potential concerns. | Strong For |  |
|  | **Education/social intervention** |  |  |
| 102 | Re-establish social contacts and social networks by arranging or provide social support via day centres. | Strong For |  |
| 103 | Education covers many specific areas: Teach patient self-management; carers (family and professional) to support self-management; teach carers to facilitate practice and/or provide care safely; encourage carers to facilitate social integration; teach patients and career disease management; and set expectations for all parties. | Strong For |  |
| 104 | An important aspect of rehabilitation after COVID-19 will be teaching patients and families how to manage the uncertainty of the disease outcomes. | Strong For |  |
| 105 | Actively share relevant knowledge about the prevention and control of COVID- with patients. | Strong For |  |
| 106 | Explain countermeasures and how to ask for help if the condition worsens. | Strong For |  |
| 107 | Include education on a healthy lifestyle and the benefits of participation in family and social activities. | Strong For |  |
| 108 | Patients with depression, anxiety, or PTSD may require psychological interventions delivered by occupational therapists, social workers, rehabilitation psychologists, or other health professionals. | Strong For |  |
| 109 | Public awareness and public education are crucial components for slowing the coronavirus spread. Providing valid and usable informative materials for people with hearing and visual disabilities (sign language translator, Braille, audio, and electronic formats) regarding the disease and infection control is a priority for several related organizations, including the American Congress of Rehabilitation Medicine. | Strong For |  |
| 110 | Educate patients and their families and/or caregivers regarding rehabilitation programs. | Strong For |  |
| 111 | Patient education: (1) Use advocacy, videos, and booklet to help patients understand the disease and treatment process; (2) advise patients to take regular rest and have a sufficient sleep; (3) to eat a balanced diet; and (4) to stop smoking. | Strong For |  |
| 112 | Provide patients with education about their condition and give strategies on how to manage recovery. | Strong For |  |
|  | **When to start rehabilitation** |  |  |
| 113 | Perform evaluation and monitoring throughout the rehabilitation period and strictly follow any indications and contraindications. | Strong For |  |
| 114 | Perform comprehensive assessments before starting the rehabilitation program. Perform clinical and exercise risk assessments based on the patient's clinical symptoms, vital signs, auxiliary examinations, imaging, comorbidities, and contraindications, whereas conduct the quality of life, daily activity endurance, and psychological and nutritional assessments, at least, in the form of questionnaires. Combine the results of these assessments with the patient's aerobic endurance, muscle strength, balance, and flexibility to formulate an individualized and progressive rehabilitation prescription. | Strong For |  |
| 115 | Ensure patients who have been infected by a coronavirus and admitted to the hospital receive respiratory physiotherapy management as soon as their condition allows. | Strong For |  |
| 116 | Consider respiratory physiotherapy interventions in hospital wards or ICU for patients who have confirmed or suspected COVID-19 and concurrently or subsequently develop exudative consolidation, mucous hypersecretion, and/or have difficulty clearing secretions. | Weak For |  |
| 117 | Provide physiotherapy interventions when there are clinical indicators to minimize staff exposure to patients with COVID-19. Note, unnecessary review of patients with COVID-19 within their isolation room/areas can have a negative impact on PPE supplies. | Strong For |  |
| 118 | Ensure to meet regularly with senior medical staff to determine indications for physiotherapy in patients with confirmed or suspected COVID-19 and screen according to set/agreed guidelines. | Weak For |  |
| 119 | Initiate respiratory rehabilitation in critically ill COVID-19 patients, once all of the following criteria are met: (1) For the respiratory system when the(i) fraction of inspired oxygen is ≤0.6, (ii) SpO2 is ≥90%, (iii) respiratory rate is ≤40 breaths/min (bpm), (iv) positive end expiratory pressure is ≤10 cmH2O (1 cmH2O = 0.098 kPa), (v) there is absence of ventilator resistance, and (vi) there is absence of unsafe hidden airway problems; (2) For the cardiovascular system when the (i) systolic blood pressure is ≥90 and ≤180 mmHg, (ii) mean arterial pressure (MAP) is between ≥65 and ≤110 mmHg, (iii) heart rate is between ≥40 and ≤120 beats/min, (iv) there is absence of new arrhythmia or myocardial ischemia, (v) there is absence of shock with lactic acid level ≥4 mmol/L, (vi) there is absence of new unstable deep vein thrombosis and pulmonary embolism, and (vii) there is absence of suspected aortic stenosis; (3) For the nervous system when the (i) Richmond Agitation-Sedation Scale score is between −2 to +2 and (ii) intracranial pressure is <20 cmH2O; and (4) others: (i) there is absence of unstable limb and spinal fractures, (ii) there is absence of severe underlying hepatic/renal disease or new progressively worsening hepatic/renal impairment, (iii) there is absence of active hemorrhage, and (iv) the temperature is ≤38.5°C. | Strong For |  |
|  | **When to discontinue rehabilitation** |  |  |
| 120 | Withdraw physiotherapy in case of high fever, worsening dyspnea, chest tightness, belching, dizziness, headache, unclear vision, palpitations, sweating, inability to keep balance, increased need for oxygen or non-invasive ventilation support, evidence of radiological lesions' progression (>50%) within 24-48 hours, and pulse oximetry are less than <93% or when at least 4-point drop during exercise (desaturation) is observed. | Strong For |  |
| 121 | Carefully monitor any changes in vital signs during rehabilitation and strictly follow rehabilitation intervention and suspension guidelines because COVID-19 can become severe in 3% to 5% of patients. | Strong For |  |
| 122 | Closely monitor patients for shortness of breath, excessive fatigue, chest pain, severe cough, blurred vision, dizziness, heart palpitations, sweating, loss of balance, decreased SaO2 (<95%), headache, or when blood pressure is <90/60 or >140/90, heart rate is >100 beats per minute, and temperature is >37.2 ºC. | Strong For |  |
| 123 | For COVID-19-positive patients, postpone the exercise program if fever, symptoms or other signs of COVID-19 infection are present. Evaluate exercise resumption on an individual basis. In general, in patients with light-to-moderate symptoms, gradually restart the exercise program after a fever-free period of 1 week and a symptom-free period of 48 h. Whenever possible, do not postpone all other cardiac rehabilitation components but provide care remotely. | Strong For |  |
| 124 | Immediately discontinue respiratory rehabilitation when one of the following conditions develops during rehabilitation: (1) dyspnea index or Borg dyspnea score is >3 (total score,10 points); (2) patient has chest tightness, shortness of breath, dizziness, headache, blurred vision, heart palpitations, profuse sweating, and balance disorder; and (3) presence of any other conditions that the clinician determines to be unsuitable for exercise. Seek assistance from physicians and nurses. | Strong For |  |
| 125 | Individual rehabilitation programs and monitored closely. Continue to stay in close communication with the primary physician of the patient about the clinical course. | Strong For |  |
| 126 | For critically ill patients, discontinue early rehabilitation immediately if any of the following conditions occur:  (1) for the respiratory system(i) SpO2 is <90% or decreases by >4% from baseline, (ii) respiratory rate is >40 bpm, (iii) ventilator resistance is required, and (iv) there is dislodgement or migration of artificial airway; (2) for the cardiovascular system when the (i) systolic blood pressure is <90 or >180 mmHg, (ii) MAP is <65 or >110 mmHg, or there is >20% change compared with baseline, (iii) when heart rate is <40 or >120 beats/min, and (iv) there is new arrhythmia, and myocardial ischemia; (3) for the nervous system when there is (i) loss of consciousness and (ii) irritability; and (4) for other conditions when there is (i) discontinuation of any treatment or removal of monitoring tube connected to the patient; (ii) patient-perceived heart palpitations, exacerbation of dyspnea or shortness of breath, and intolerable fatigue; and (iii) falls in the patient. | Strong For |  |
| 127 | Carry out all under the premise of safety. In case a patient shows peripheral capillary oxygen saturation (SpO2 is< 88%) or develops symptoms, such as palpitations, sweating, chest tightness, and shortness of breath, which are considered unsuitable for rehabilitation by the clinician, terminate the rehabilitation program immediately. | Strong For |  |
|  | **Special populations** |  |  |
| 128 | Ensure Comprehensive Geriatric Assessment (CGA) for older adults with rehabilitative needs remains a priority. CGA includes interprofessional geriatric assessment data, physical assessment findings, analysis and synthesis of the clinical profile, and development of a collaborative plan and follow-up plan of care. | Strong For |  |
| 129 | Ensure an interdisciplinary approach for rehabilitative care for older adults  Develop capacity/contingency plans by partnering with organizations and/or regions to ensure interdisciplinary teams with skills and knowledge in geriatrics are available to deliver care across the continuum. | Strong For |  |
| 130 | Include older adults and their caregivers as part of the team approach. Caregivers and family members have an integral role, and their impact in providing physical, social, emotional, and navigation of care is crucial among COVID-19 restrictions. | Strong For |  |
| 131 | Take into account health problems beyond chronological age. For example, consider the presence or absence of frailty, functional status, and multi-multimorbidity as predictors for complications in older adults with acute illnesses. | Strong For |  |
| 132 | Discuss prehabilitation with the medically frail or at-risk patient. Prehabilitation involves medical interventions aimed at preventing or reducing the severity of physical impairments in anticipation of a physical stressor. Examples include cancer or surgical prehabilitation to improve treatment-related morbidity and mortality, and psychological health outcomes. Delivery of home-based programs to proactively monitor vulnerable populations via telemedicine. | Strong For |  |
| 133 | Rehabilitative care is essential to prevent functional decline and disability in older adults. Support older adults to optimize their functional ability, engage in meaningful activity and maintain independence. | Strong For |  |
| 134 | Accommodate differing levels of tolerance of rehabilitative care for older adults. Adjust services as the individual's tolerance level changes to achieve patient goals. | Strong For |  |
| 135 | Have the provider determine the most appropriate level of rehabilitative care (prevention, stabilization, progression, and/or maintenance) based on individual patient needs and recognizing needs may fluctuate along the course of the functional/recovery trajectory.  Use the Referral Decision Tree for Rehabilitative Care and Definitions Frameworks (levels of care and community-based) guidance to determine the appropriate level of care. The stratification of the rehabilitation need can prevent a particular rehabilitation setting from being overwhelmed when the COVID-19 pandemic wave moves from acute to post-acute care. | Strong For |  |
| 136 | Provide multimodal education to patients and caregivers. Tailor materials to an individual's preferences and experiences while considering the person's level of health literacy. | Strong For |  |
| 137 | Develop and approve transition plans in partnership with the patient, any caregivers involved, and the care team. Ensure caregivers provide essential support to coordinate care at major transition times, particularly during the COVID-19 pandemic due to potential reductions in or changes in the community-based health service delivery. | Strong For |  |
| 138 | Ensure timely access to rehabilitation programs and services for older adults (e.g., inpatient, outpatient, community-based, etc.) to minimize adverse outcomes for vulnerable older adults and support those who may have initial states of functional decline. | Strong For |  |
| 139 | Recommend proactive case finding to identify older adults requiring rehabilitative care. Screening approaches are critical, particularly in primary care and community settings. Fear of COVID-19 transmission from interactions within health care facilities or through visits with health care providers may deter older adults with impaired function from not being present until a point of increased frailty or illness. Ensure older adults are provided with post-acute care navigation to appropriate rehabilitative services. | Weak For |  |
| 140 | Community-based services available to older adults vary. To ensure optimal care and longer-term management, liaise with local community services to determine the best model of care for the frail senior, such as primary care teams, home and community care, or specialized geriatric services. | Strong For |  |
| 141 | Recognize that caregivers are essential to the care of frail seniors and are key in many settings to the provision of care. Caregivers often serve as liaisons between patients and clinicians and are involved in day-to-day decision-making and care delivery. Ensure, therefore, to include caregivers in the healthcare team's communication and care planning. Additionally, provide caregivers access to necessary resources. In the context of the pandemic, provide caregivers with personal protective equipment (PPE) with instructions on proper donning and doffing techniques. | Strong For |  |
| 142 | Have health care providers provide advice about when it is safe to exercise to children, older adults, and those who have previously experienced symptoms of illness or those susceptible to chronic cardiovascular or pulmonary disease. | Strong For |  |
| 143 | Consider approaches to stratify rehabilitation levels of care for older adults within each local context, given anticipated increases in demand for rehabilitation allow for the right mix of skills to be deployed by matching needs in rehabilitative care with the right level of care. | Strong For |  |
| 144 | The anticipated increase in demand for rehabilitation requires an increase in capacity. Alternate delivery models such as virtual rehabilitative care options or rehabilitation services offered in alternate infrastructures during recovery phases may be needed to accommodate the growing number of older adults who require inpatient rehabilitation. | Strong For |  |
| 145 | A more diverse rehabilitation workforce may also be necessary to meet the scale of this challenge. As staff-to-patient ratios potentially decrease due to a reduction in group programming (notwithstanding virtual care options), increasing the capacity of a rehabilitative care workforce may become essential. | Strong For |  |
| 146 | Consider moving rehabilitative care for older adults beyond bedded rehabilitation settings to put services in primary care and the community at the forefront and engineer better collaboration with organizations across the continuum of care. Consider delivering care closer to home, and to be effective, ensure teams work across organizational boundaries and not in silos. | Strong For |  |
| 147 | Have Physical therapists (PTs) provide help to older adults during pandemic from home by utilizing online internet technologies to add a social element to exercise performance for those who get depressed or bored during lonely-home training. | Strong For |  |
| 148 | Have PTs generate different home-based exercises, including simple resistance, strengthening, stretching, and breathing, via scheduled live videos and diagrams via social media for older adults, their caregivers and their families. | Strong For |  |
| 149 | Recommend a multicomponent exercise and adequate program consisting of aerobic, resistance, balance, coordination, and mobility training exercises for older adults residing in free-living and community-dwelling settings. | Strong For |  |
| 150 | Use the following international guidelines of physical activity for older people: (1) recommend exercise frequency of 5 days per week, in quarantine situation exercise, can be increased to 5-7 days per week with adaptation in volume and intensity. (2) recommend exercise volume of at least 150 to 300 minutes per week of aerobic exercise and two resistance training sessions per week. When in quarantine, exercise duration can be increased to 200-400 minutes per week and distributed among 5-7 days to compensate for the decrease in the normal daily physical activity levels. Moreover, recommend a minimum of 2-3 days per week of resistance exercise. Suggest supplementing with mobility training exercises on all the training days and balance and coordination exercises on different training days (at least twice).  (3) recommend moderate exercise intensity for most of the sessions with some vigorous exercise per week. Reinforce the fact moderate intensity improves the immune system while vigorous intensity may inhibit the immune system, particularly in sedentary people. Thus, during quarantine, ensure moderate intensity achieving (40-60% heart rate reserve or 65-75% of maximal heart rate) is the ideal choice for older people to enhance the protective role of exercise. | Strong For |  |
| 151 | The following measures are to reduce the risk of introduction of SARS-CoV-2 in institutions caring for older adults and to reduce the risk of nosocomial transmission:  - Consider lockdowns, suspension of visits and personal aids, securement of supply chains, isolation of cases, an extension of barrier measures, sanitation, and limiting internal activities.  - Outline coping approaches for institutions with a double burden; a high burden of disease among residents with constraints of severe staff.  -Public information and communication campaigns to protect the most vulnerable and oldest people of our society, to make them visible, and to provide strong psychological support to the nursing staff.  - Strengthen the communication between nursing staff and families at the end of a resident's life as well as after death.  - to offset the burden on general hospitals, offer a palliative care approach grounded in ethics within the impacted institutions. | Strong For |  |
| 152 | Transition physiatrist outpatient in-person visit to virtual physical exams (telemedicine) that can be delivered using virtual workflow (before, during, and after the visit) during natural disasters or the pandemic. | Strong For |  |
| 153 | Ensure participation in taijiquan, wuqinxi, baduanjin, and other physical activities to regulate the breath, dredge meridians, and improve stability of the core muscle group and balance ability. These activities are particularly suitable for older adults or weak patients with low physical abilities that can be carried out by mild, medium, and discharged patients, using group mutual aid mode or while at home through video exercises. | Strong For |  |
| 154 | The World Health Organization guidelines for supports and services for persons with disabilities include having Psychiatrists provide education on the increased risk of the novel coronavirus, basic protection measures against the virus to mitigate the risk. These measures include 90-day mail-order prescriptions, ordering extra supplies for home care, help in the identification of accessible home health agencies, and screening for exacerbation of mental health conditions. | Weak For |  |
